# Quercetin-solid lipid nanoparticle-embedded hyaluronic acid functionalized hydrogel for immunomodulation to promote bone reconstruction

**DOI:** 10.1093/rb/rbad025

**Published:** 2023-04-11

**Authors:** Pinghui Zhou, Bomin Yan, Bangguo Wei, Liangmin Fu, Ying Wang, Wenrui Wang, Li Zhang, Yingji Mao

**Affiliations:** Department of Orthopaedics, The First Affiliated Hospital of Bengbu Medical College, Bengbu 233004, China; Anhui Province Key Laboratory of Tissue Transplantation, Bengbu Medical College, Bengbu 233030, China; Department of Plastic Surgery, The First Affiliated Hospital of Bengbu Medical College, Bengbu 233004, China; Department of Orthopaedics, The First Affiliated Hospital of Bengbu Medical College, Bengbu 233004, China; Department of Plastic Surgery, The First Affiliated Hospital of Bengbu Medical College, Bengbu 233004, China; Department of Orthopaedics, The First Affiliated Hospital of Bengbu Medical College, Bengbu 233004, China; Department of Plastic Surgery, The First Affiliated Hospital of Bengbu Medical College, Bengbu 233004, China; Department of Orthopaedics, The First Affiliated Hospital of Bengbu Medical College, Bengbu 233004, China; Department of Plastic Surgery, The First Affiliated Hospital of Bengbu Medical College, Bengbu 233004, China; Department of Orthopaedics, The First Affiliated Hospital of Bengbu Medical College, Bengbu 233004, China; Department of Plastic Surgery, The First Affiliated Hospital of Bengbu Medical College, Bengbu 233004, China; School of Life Science, Bengbu Medical College, Bengbu 233030, China; Anhui Province Key Laboratory of Translational Cancer Research, Bengbu Medical University, Anhui 233030, China; Department of Orthopaedics, The First Affiliated Hospital of Bengbu Medical College, Bengbu 233004, China; Anhui Province Key Laboratory of Tissue Transplantation, Bengbu Medical College, Bengbu 233030, China; Department of Plastic Surgery, The First Affiliated Hospital of Bengbu Medical College, Bengbu 233004, China; Department of Orthopaedics, The First Affiliated Hospital of Bengbu Medical College, Bengbu 233004, China; School of Life Science, Bengbu Medical College, Bengbu 233030, China; Anhui Province Key Laboratory of Tissue Transplantation, Bengbu Medical College, Bengbu 233030, China; Department of Plastic Surgery, The First Affiliated Hospital of Bengbu Medical College, Bengbu 233004, China

**Keywords:** quercetin, hydrogels, macrophages, inflammatory microenvironments, bone tissue engineering

## Abstract

Bone defects are a persistent challenge in clinical practice. Although repair therapies based on tissue-engineered materials, which are known to have a crucial role in defective bone regeneration, have gathered increased attention, the current treatments for massive bone defects have several limitations. In the present study, based on the immunomodulatory inflammatory microenvironment properties of quercetin, we encapsulated quercetin-solid lipid nanoparticles (SLNs) in a hydrogel. Temperature-responsive poly(ε-caprolactone-co-lactide)-b-poly(ethylene glycol)-b-poly(ε-caprolactone-co-lactide) modifications were coupled to the main chain of hyaluronic acid hydrogel, constructing a novel, injectable bone immunomodulatory hydrogel scaffold. Extensive *in vitro* and *in vivo* data showed that this bone immunomodulatory scaffold forms an anti-inflammatory microenvironment by decreasing M1 polarization, while elevating the M2 polarization. Synergistic effects on angiogenesis and anti-osteoclastic differentiation were observed. These findings further proved that administering quercetin SLNs encapsulated in a hydrogel can aid bone defect reconstruction in rats, providing new insights for large-scale bone defect repair.

## Graphical Abstract

**Figure:**
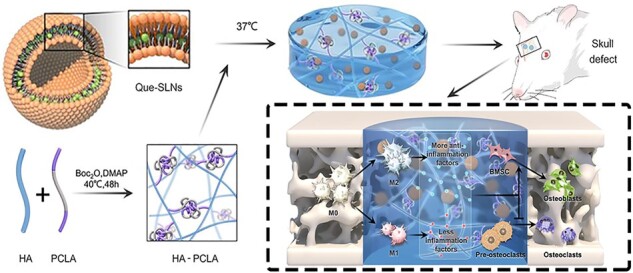

## Introduction

Large-scale bone defects are a challenge in clinical practice owing to the difficulty of self-healing, as the severity of the defect is beyond the compensatory range of the bone tissue. The current gold standard for treating massive bone defects is autologous or allogeneic bone grafting [1–3]. It is restricted by the limited availability of tissue at the donor site, and the potential impact on the donor site after transplantation often fails to meet medical needs [4–6]. Consequently, repair therapies based on tissue-engineered materials have emerged as a popular research topic [7–9]. However, the effectiveness of bone repair with tissue-engineered materials can be negatively affected by the foreign body reaction and long-term inflammatory response of the material to the contact surface of the implant site [4, 10, 11]. An effective approach is urgently required to address this challenge.

Bone immunomodulation has attracted considerable attention as a way to shape scaffolds with favorable immunomodulatory properties. Scaffolds are implanted to activate the body’s immune system and regulate the immune microenvironment at the site of bone defects [12, 13]. This suppresses inflammation and secretes factors that favor bone repair, enhancing bone regeneration [14]. Bone repair is a multi-step process. During bone regeneration, the inflammatory response phase initiates early and significantly influences the subsequent angiogenic and osteogenic differentiation responses [15, 16]. Natural immune cells mediate the inflammatory response; macrophages are one of the first cells to be recruited to the implant surface and are essential for the regulation of the bone immune response [17, 18].

Macrophages exhibit great plasticity and can differentiate as different phenotypes when responding to various stimuli. This results in different efficacies and is known as macrophage polarization. Resting macrophages (M0) are mainly polarized into two phenotypes: M1 and M2 [19, 20]. The M1 phenotype is mainly characterized by the production of pro-inflammatory cytokines, such as IL-1β and tumor necrosis factor-alpha (TNF-α) [18, 21, 22]. These pro-inflammatory signals play a role in stimulating osteoclast responses and directing osteogenic differentiation responses [23]. Nevertheless, long-term pro-inflammatory factor stimulation leads to chronic inflammation at the defect site, which is detrimental to bone repair [14]. Comparatively, M2 macrophages suppress inflammation by producing anti-inflammatory factors, mainly IL-4 and IL-10, and M2 macrophages can recruit mesenchymal stem cells (MSCs) by secreting signals, such as vascular endothelial growth factor (VEGF) and bone morphogenetic protein (BMP-2), to facilitate angiogenesis and induce bone differentiation [15, 20]. Therefore, regulating the polarization of macrophages toward the major M2 phenotype can help counteract the inflammation generated on the surface of the implanted material [24] and create an ideal immune microenvironment for the repair of bone defects and promote bone regeneration [25–27]. Researchers have adopted several different protocols to modulate the macrophage phenotype and the immune response, mainly involving the encapsulation of anti-inflammatory drug components, or relevant biological factors in tissue-engineered scaffolds, or the incorporation of extracellular matrix (ECM) or cells to modulate inflammation [24, 28]. Botanical extract drugs are emerging as products of interest owing to their natural immunomodulatory mechanisms and low toxicity. Various botanical extracts, such as resveratrol and curcumin, are being investigated to modulate macrophage polarization [29, 30], and the construction of plant-bearing tissue engineering scaffolds for the repair of massive bone defects is beginning to be a feasible direction that offers immense potential.

Quercetin (Que) is a low-cost and low-risk plant extract drug with few side effects, and its anti-inflammatory and immunomodulatory roles are gaining increasing attention [14, 31]. Recent studies showed that Que significantly reduces the levels of macrophage M1 polarization markers, such as IL-1β, IL-6 and TNF-α, by inhibiting the NF-κB pathway and enhancing M2 polarization levels by activating the AMPK and Akt signaling pathways, resulting in remarkable anti-inflammatory effects [32–34]. In recent years, Que was effective in the immunomodulatory repair of periodontitis, pulmonary inflammation and acute kidney injury inflammation [32, 33, 35]. It may play a vital role in anti-inflammatory responses. The osteogenic ability of Que also comes from aspects such as the inhibition of osteoclast formation through the RANKL pathway, and its capacity to promote bone regeneration [36]. However, it is a poorly water-soluble flavonoid [37], which poses a barrier to its absorption *in vivo*. Certain solid lipid nanoparticles (SLNs) facilitate solute uptake *in vivo* [38–40] and have well-controlled drug release *in vivo,* with excellent biocompatibility and structure-assisted drug uptake [41–43]. In our previous work, we loaded resveratrol into SLN scaffolds to repair bone defects. These scaffolds exhibited slow and sustained drug release to facilitate osteogenic differentiation and bone regeneration [44].

In this study, we attempted to enhance the immunomodulatory effect of Que on macrophages to form an immune microenvironment conducive to bone regeneration by formulating a new composite hydrogel using SLNs encapsulated with Que and uniformly loaded Que-SLNs into the hydrogel support. We selected hyaluronic acid—poly(ε-caprolactone-co-lactide)-b-poly(ethylene glycol)-b-poly(ε-caprolactone-co-lactide) hydrogel (HA-PCLA hydrogel) as the base hydrogel because of its low toxicity, high biocompatibility, and bioresorbability. The HA component is one of the most important and abundant components of the ECM that is accessible, and its acidic functional groups facilitate enhanced *in situ* biomineralization [45].

**Figure 1.**
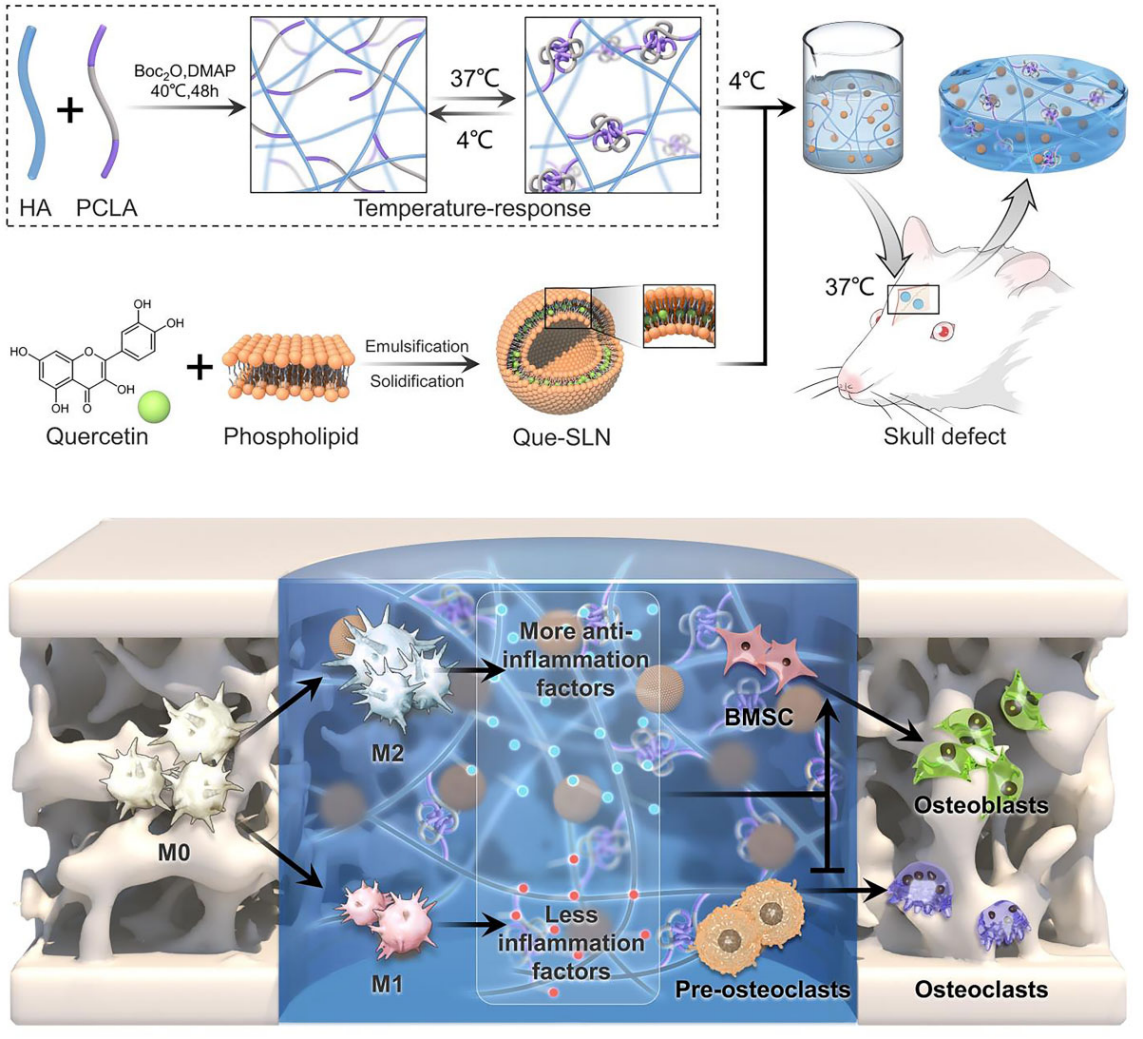
*In situ* bone repair by Que-SLNs@PCLA-HA in a rat calvarial defect model and its macrophage immunoregulatory mechanism.

In summary, we loaded Que into SLNs (Que-SLNs) and homogeneously packed them into HA-PCLA hydrogel scaffolds. This scaffold induced osteogenic differentiation, while enhancing the immunomodulatory effect on macrophages, to shape an immune microenvironment favorable for bone regeneration. Extensive *in vitro* experiments were conducted to confirm the osteogenic properties of the scaffold, and rat models with critical-sized cranial defects were used to explore the osteogenic repair ability of the hydrogel scaffold (Fig. 1). The Que-SLN@HA-PCLA hydrogel is a promising alternative material for bone repair.

## Materials and methods

### Synthesis of Que-SLNs

Synthesis of Que-SLNs was performed using the solvent emulsion diffusion method [35] (refer the Supplementary Material for details).

### Characterization of Que-SLNs

Particle characterization included transmission electron microscopy (TEM), particle size and Zeta potential measurements, X-ray diffraction (XRD), Fourier transform infrared spectroscopy (FTIR), incorporation efficiency and drug loading efficiency (for details, refer the Supplementary Material).

### Preparation and characterization of Que-SLN@HA-PCLA scaffolds

The synthesis of HA-PCLA was performed as previously reported [46] (refer the Supplementary Material and Supplementary Fig. S1 for details). HA-PCLA copolymer solution was prepared at a concentration of 15 wt.% by using deionized water as the solvent. The HA-PCLA polymer exhibits a sol–gel phase transition between room temperature and body temperature, exists in the sol state at 4–10°C, and converts to the gel state at 37°C [46, 47]. The Que-SLN@HA-PCLA hydrogels encapsulated with the corresponding lipid nanoparticles were prepared by uniformly mixing Que-SLN particles in HA-PCLA sol at 4–10°C and then gelling the mixture with a water bath at 37°C.

The Que-SLN@HA-PCLA scaffolds were synthesized by mixing different mass fractions of Que-SLNs (0.005, 0.01, 0.02 and 0.04) with soluble HA-PCLA. Therefore, the following Que-SLN@HA-PCLA hydrogel scaffolds were used to conduct the subsequent experiments: HA-PCLA, SLN@HA-PCLA, 0.005 Que-SLN@HA-PCLA, 0.01 Que-SLN@HA-PCLA, 0.02 Que-SLN@HA-PCLA and 0.04 Que-SLN@HA-PCLA.

### Characterization of Que-SLN@HA-PCLA scaffolds

Characterization of the Que-SLN@HA-PCLA scaffolds was performed by nuclear magnetic resonance (NMR), FTIR, scanning electron microscopy (SEM), drug release and hydrogel degradation *in vitro*. Refer the Supplementary Material for more details on the hydrogel characterization of the Que-SLN@HA-PCLA scaffolds.

### Viability of BMSCs on different scaffolds

Detailed descriptions of the isolation and culture of bone marrow MSCs (BMSCs) are presented in the Supplementary Material. Live/dead staining assay kits were used to detect cell viability. Classical six-well culture plates were coated with 1 ml Que-SLN@HA-PCLA hydrogels at different concentrations per well. Equal amounts of pure HA-PCLA and hydrogels of SLN@HA-PCLA without Que addition were used as controls. Bone marrow stromal stem cells were grown on their surface at a density of 1 × 10^5^/well. Live/dead cells in the samples were stained at the time of cell culture, Days 1 and 3 using the Calcein-AM/propidium iodide (PI) double staining kit (Yeasen, China) according to the manufacturer’s instructions. Next, the cells were analyzed using an environmental scanning electron microscope (QUANTA250, USA).

### Osteogenesis measurement of BMSCs on Que-SLNs@HA-PCLA scaffolds

#### Alkaline phosphatase activity

Alkaline phosphatase is widely distributed in mammalian tissues and commonly used to identify early markers of bone formation. We subjected the hydrogel scaffolds to grouping and dosage as described in the sections above. The culture was incubated for 7 days at 37°C in an osteogenesis induction medium (OM, Cyagen, China), followed by ALP staining with an ALP staining kit (Beyotime, Shanghai, China). The cells were fixed in paraformaldehyde (4%), followed by staining with 5-bromo-4-chloro-3-indolyl phosphate/nitro blue tetrazolium (BCIP/NBT) in the dark for 15 min at 4°C. Excess BCIP/NBT reagent was removed, and blue ALP-positive cells were observed under an inverted microscope (Zeiss Axio Observer Z1, Germany).

#### Alizarin red staining

Alizarin red staining (ARS) (Suzhou Yongqinquan Intelligent Equipment Co. Ltd., China) is commonly used to detect mineralized osteoblast nodules [48]. Cells were cultured for 14 days in a 24-well plate, the cells were fixed with 4% paraformaldehyde for 20 min and stained with 2% alizarin red for 5 min at 4°C. The plates were removed and analyzed using an inverted microscope.

#### Immunofluorescence staining

Osteocalcin (OCN) is a typical late-stage marker involved in osteogenic differentiation. The hydrogel scaffold and dosage were the same as described above. Briefly, BMSCs were cultured at 2 × 10^4^ cells/ml in 24-well plates for 14 days at 37°C, followed by fixation with 4% v/v paraformaldehyde, permeabilization with 0.3% v/v Triton X-100 and blocking with 1% w/v bovine serum albumin. The cells were incubated with 5 μg/ml OCN primary antibody (1:10, Affinity, DF12303, China) at 4°C overnight and stained with Cy3-labeled secondary antibody (1:200, Affinity, S0011, China) in the dark for 2 h at 4°C. The nuclei were stained with 5 μg/ml 4′,6-diamidino-2-phenylindole (DAPI) for 5 min at 4°C and observed using an inverted fluorescence microscope (Zeiss Axio Observer Z1, Germany).

RAW264.7 cells from the Type Culture Collection of the Chinese Academy of Sciences were stimulated and treated with different concentrations of Que scaffolds to mimic the changes in the immune microenvironment during defective bone reconstruction. This was done to investigate how different concentrations of Que scaffolds affect macrophage polarization. The distribution of M1 and M2 phenotype macrophages was subsequently probed by immunofluorescence: RAW264.7 macrophages were cultured using DMEM complete medium with 10% FBS, counted, and the cell suspension was inoculated onto Que hydrogel scaffolds at different concentrations at a density of 8 × 10^5^ cells/well in a 24-well plate and incubated for 48 h at 37°C. Immunofluorescence staining of different scaffold groups was performed using inducible nitric oxide synthase (iNOS, M1 phenotype marker, 1:10, Affinity, AF0199) and arginase-1 (Arg-1, M2 phenotype marker, 1:10, Affinity, DF6657) as primary antibodies, then stained with Cy3-labeled secondary antibody (1:200, Affinity, S0011, China) and FITC-labeled secondary antibody (1:200, Affinity, S0007, China), respectively, in the dark for 2 h at 4°C. The nuclei were stained with DAPI. The corresponding fluorescence images were obtained using fluorescence microscopy (Zeiss Axio Observer Z1, Germany) under different fluorescence excitations.

#### Real-time PCR analysis

The osteogenic culture was performed for 14 days using the same method as described above. Total RNA was extracted from bone marrow MSCs using TRIzol reagent, and cDNA was synthesized using the HiScript II Q RT SuperMix kit (Vazyme, China). The relative mRNA expression was normalized and calculated using the 2^−ΔΔCT^ method [49]. The expression of osteogenesis-related Alp, OCN, Runx2, OPN, Col-1 and OSX was examined, and all the gene-specific primers used in this experiment are shown in Supplementary Table S1.

### Effects of different hydrogel scaffolds on bone regeneration *in vivo*

#### Establishment of cranial defect model in rats

All animal experiments were performed in compliance with the National Research Council’s Guide for the Care and Use of Laboratory Animals and were approved by the Ethics Committee of the Medical Faculty of Bengbu Medical College (approval number: 2021272). Six-week-old male Sprague Dawley (SD) rats were purchased from the Shushan Laboratory Animal Centre, Hefei, China. The rats were housed in a quiet, enclosed room with a 12-h light-dark cycle for 7 days before surgery and fasted for half a day to ensure the effectiveness of anesthesia. Rats were intraperitoneally injected with 0.3 ml 10% chloral hydrate solution per 100 g weight as an anesthetic agent. Cranial defect in rats was generated by using the skull drilling method to create 5 mm symmetrical total skull defects on both sides of the midline of the rat cranial, as detailed in the Supplementary Material. Prefabricated scaffolds of different groups carrying the same mass of 100 mg (HA-PCLA, SLN@HA-PCLA, Que@HA-PCLA and Que-SLN@HA-PCLA) were implanted in the defects. Among them, the Que-SLN quality fractions in Que-SLN@HA-PCLA were taken as 0.01 of the best results achieved in the previous *in vitro* experiments, consistent with Que drug content in the Que@HA-PCLA group. The rat skin was subcutaneously sutured after implantation. Rats were divided into a cranial defect model without scaffold implantation as a blank control group. The rat tails were marked with their grouping, and the rats were mixed and randomly selected for rearing in the cages. Penicillin was injected (40 000 daily units) for 3 days after surgery to fight the infection.

#### Micro-computed tomography measurement

The rats were euthanized postoperatively after 4 and 8 weeks. Skull specimens were collected at the hydrogel implantation site, immobilized in 4% paraformaldehyde for 24 h and scanned by micro-computed tomography (SkyScan 1176, Belgium) at the site of the skull defect in the rat head using the following settings: 65 kV, 385 mA and 1 mm aluminum filter. A 3D reconstruction was performed using simulation software. The designated cylindrical region of interest containing the defect was selected, and the bone volume (BV) fraction (BV/tissue volume, BV/TV) and bone mineral density (BMD) were calculated using a CT analyzer (SkyScan, Belgium).

### Histological analysis

#### Hematoxylin–eosin staining and Masson’s staining

Cranial specimens were obtained as described above and decalcified using EDTA decalcification solution (pH 7.2) for 30 days. Specimens were routinely dehydrated and embedded in paraffin. A 5-μm-thick section was sliced from the center of the hydrogel implantation area in each group of specimens by using a slicing machine (Leica RM2125RTS, Germany), and the resulting sections were stained with hematoxylin–eosin (H&E) and Masson's trichrome reagent. The specimens were observed under a light microscope to assess bone formation.

#### Tartrate-resistant acid phosphatase staining

Tartrate-resistant acid phosphatase (TRAP) staining was performed to detect osteoblast-like cells. Briefly, TRAP solution (Servicebio, G1050-50T, China) was prepared by mixing 40 mM sodium acetate, 50 mM sodium tartrate, naphthol AS-MX phosphate and N,N-dimethylformamide. The sections were stained with solid red violet LB dissolved in TRAP solution for 90 min at 37°C. Nuclei were simultaneously stained and counterstained with diaminobenzidine and hematoxylin.

#### Immunohistochemical analysis

The sections were dewaxed, hydrated and placed in citrate buffer (0.5 M, pH 5.0) for antigen extraction. The sections were exposed to 3% methanol/H_2_O_2_ and endogenous peroxidase was blocked with 3% w/v BSA. The sections were incubated overnight with anti-OCN (1:100, Affinity, DF12303) and anti-Runx2 (1:100, Affinity, AF5186) primary antibodies, then incubated with horseradish peroxidase-coupled secondary antibodies (1:200, S0001, Affinity). Nuclei were stained and counterstained with diaminobenzidine and hematoxylin.

The gene expression of pro-inflammatory factors IL-1β (1:100, Affinity, AF5103) and IL-4 (1:100, Affinity, AF5142) was analyzed to detect the inflammatory mechanism of the scaffold's ability to promote bone regeneration.

#### Immunofluorescence staining

Immunofluorescence staining of CD31 provides a promising evaluation of the formation of new capillaries in the tissue and the rate of deposition of new bone [50]. Briefly, sections were incubated overnight at 4°C with a primary antibody (1:200, Affinity, AF61911) followed by incubation with Cy3-coupled goat anti-rabbit secondary antibody (1:200, Affinity, S0011) for 1 h at 37°C. Finally, the nuclei were stained with DAPI (Solarbio, China). Images were captured with a fluorescence microscope and analyzed using Image-Pro Plus software (version 7.0).

The percentage of macrophages with different phenotypes and differentiation was determined. Briefly, M1-type macrophages were labeled with a CCR7 antibody (1:100, Affinity, AF5293) and incubated overnight at 4°C with the Alexa 488 donkey anti-rabbit IgG secondary antibody (1:200, Yeasen, 34206ES60, China). Likewise, M2 macrophages were labeled with CD206 antibody (1:100, Thermo Fisher, PA5-46994). The nuclei were stained with DAPI. The images by fluorescent microscopy were captured and analyzed with Image-Pro Plus software (version 7.0).

### Statistical analysis

All data are expressed as the mean ± standard deviation. Statistical significance was calculated with the use of ANOVA (analysis of variance) and Student’s *t*-test to compare statistical significance. The statistical significance of the differences between groups was analyzed using one-way ANOVA followed by Tukey’s post hoc variance test. Each group was compared with the control group. Statistical significance was set at *P *<* *0.05.

## Results

### Characterization of Que-loaded SLNs

**Figure 2.**
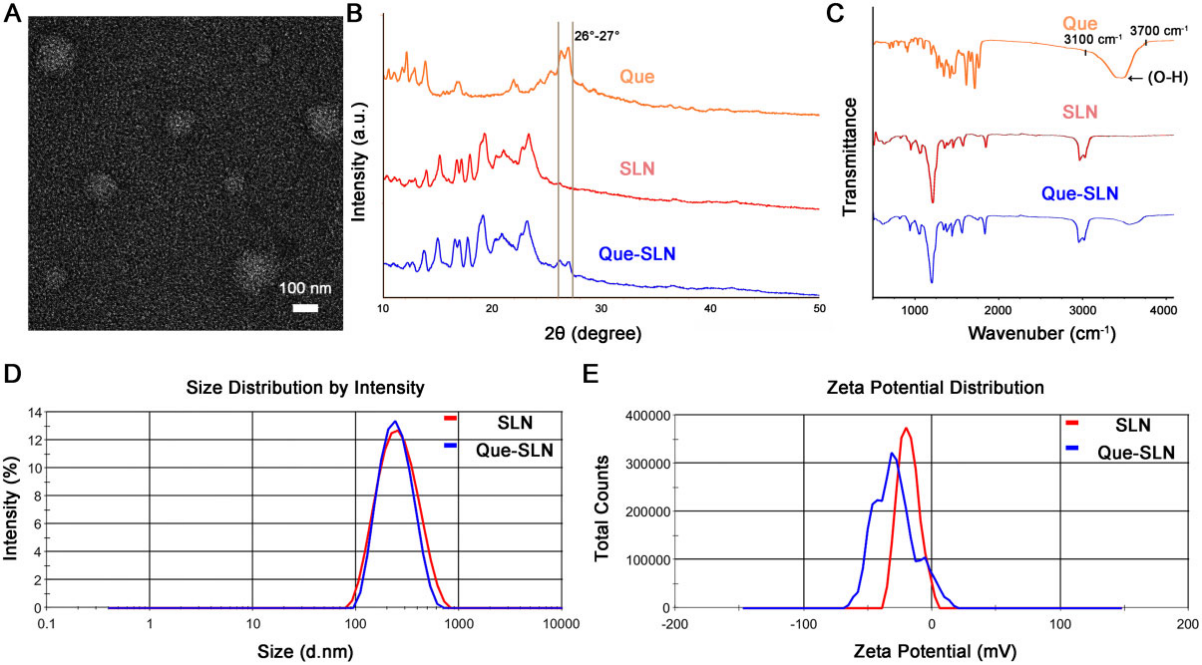
Morphology and characterization of Que-SLNs. (**A**) Morphology of Que-SLN particles observed by TEM. (**B**, **C**) XRD and FTIR spectra of que sample particles, SLN sample particles and Que-SLN particles, respectively. (**D**) Particle size analysis and (**E**) zeta potential analysis of SLN sample particles and Que-SLN particles.

All prepared Que-SLNs were loaded with Que onto SLNs in strict accordance with the solvent diffusion method. For the delicate observation of the structure of Que-SLN on the nanoscale, 2% phosphotungstic acid was used for negative staining and TEM was performed. Since this dye has difficulty penetrating the bilayer vesicle structure of SLN, the white coloring of SLN against the dark background can be distinctly observed. The TEM nanoscopic images demonstrated that the Que-SLNs were in the morphology of spherical particles with an average particle size of 100–200 nm (Fig. 2A). Furthermore, the uniformity of the overall nanoparticle morphology and the absence of aggregation signified that the loading of Que had no significant impact on the topography and stabilization of the nanoparticles.

The X-ray powder diffractograms of the synthesized Que-SLNs (Fig. 2B) displayed several different diffraction peaks. This indicates that their structure was highly crystalline. Que-SLNs had a distinct double absorption peak in the vicinity of 26°–27° with high similarity to Que which was not observed in blank SLNs. This illustrated that Que was successfully loaded in the SLNs. Interpretation of the FTIR spectra further revealed the molecular structure and properties of the Que-SLNs (Fig. 2C). The characteristic alkane-containing C–H extended absorption peaks (3000–2850 cm^−1^) of SLNs were detected in the FTIR spectra of Que-SLNs. This indicated that Que-loading did not disturb the original structure of SLNs. Moreover, the Que-SLN FTIR spectra revealed a characteristic O–H absorption peak of Que (3700–3100 cm^−1^) [51]; however, this peak was not detected in the blank SLNs. This further indicated that the Que component was physically encapsulated in the SLNs.

Precise determination of the particle size and Zeta potential was employed to investigate the physical morphology and particle stability of the SLNs caused by Que loading. The average diameters of blank SLNs and Que-SLNs were 118.3 ± 22.3 nm (polydispersity index = 0.35 ± 0.01) and 128.1 ± 27.2 nm (polydispersity index = 0.26 ± 0.01), respectively, with only a single-peak distribution (Fig. 2D). This indicated that Que loading had insignificant effects on the morphology of SLNs with favorable homogeneity; this data correlated with the TEM results. The Zeta potential of Que-SLNs (−30.2 ± 0.9 mV) is close to that of SLNs (−33.2 ± 0.7 mV) (Fig. 2E). This also confirms that Que loading did not have a remarkable impact on the particle stability of SLNs. The Que entrapment efficiency in the particles of Que-SLNs was 80.2 ± 3.3% according to the HPLC results.

### Characterization of Que-SLNs loaded composite hydrogel

**Figure 3.**
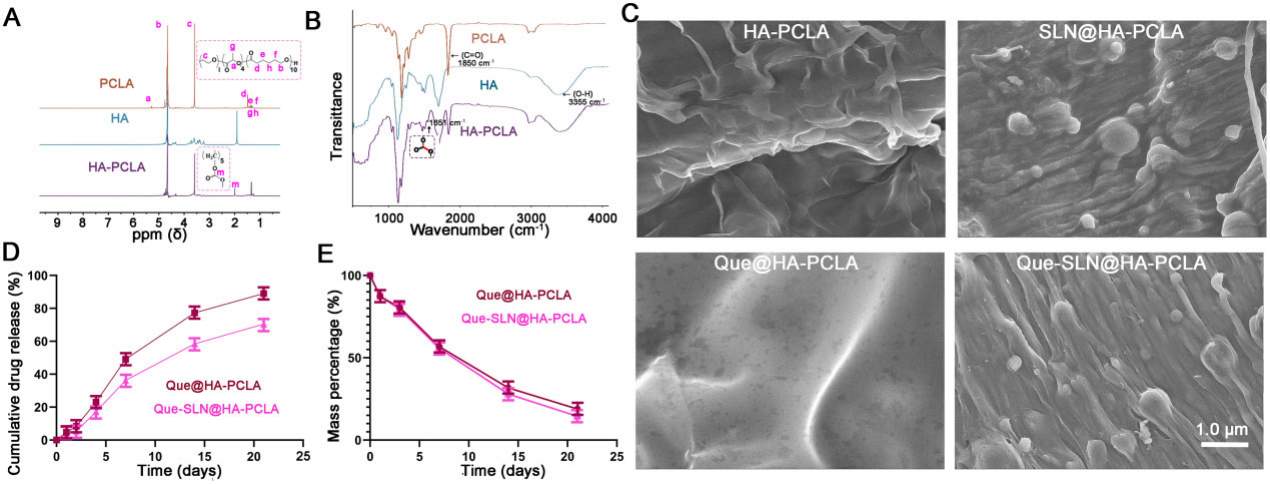
Que-SLN@HA-PCLA Morphology and characterization of the hydrogel. (**A**, **B**) NMR and FTIR spectroscopy analyses of PCLA, HA and HA-PCLA hydrogel scaffolds, respectively. (**C**) Different morphology of hydrogel scaffolds observed under SEM. (**D**) Drug release rates and (**E**) degradation of Que-SLN@HA-PCLA hydrogels *in vitro*.

The HA-PCLA polymer was synthesized by esterification of PCLA conjugated with HA, wherein the PCLA polymer was fabricated by ring-opening polymerization of ε-caprolactone (CL) and propylene glycerides (LA). The NMR spectral results showing characteristic aliphatic peaks at 1.25–1.52 ppm in the CL and 4.94 ppm in the LA acrylate-glyceride unit in the PCLA spectra substantiated the combination of CL and LA into PCLA polymers (Fig. 3A). The characteristic peak of the HA sugar ring at 3.13–4.48 ppm in the spectrum of the HA-PCLA polymer revealed that the HA component was incorporated with the polymer. The aliphatic peak at 1.25–1.65 ppm and the CL acetyl peak at 2.09 ppm confirmed the binding of PCLA with HA by esterification [52, 53].

The interpretation of the FTIR results further revealed the bonding of the molecular structures in the hydrogel (Fig. 3B). The same characteristic peaks of propyl cross-ester C–O and polycaprolactone C=O in PCLA were identified at 1210 and 1850 cm^−1^ in the HA-PCLA spectrum [54–56], whereas the characteristic absorption peaks of HA-related C–O–C, C=O and O–H emerged at 1151, 1650 and 3355 cm^−1^, respectively, in the HA-PCLA spectrum [57]. Also, the strong absorption peak at 1651 cm^−1^ was attributed to the newly generated ester bond, which further confirmed the successful binding of HA to PCLA.

SEM images showed that the HA-PCLA hydrogels featured a smooth surface and uniform thickness (Fig. 3C). The hydrogels were wrapped around the SLNs and formed spherical solid particles with a homogeneous morphological distribution when loaded with SLNs. The addition of Que did not result in a morphological discrepancy compared with the group lacking Que. This implied that the loading of Que did not significantly affect the original structure of HA-PCLA hydrogels.

### Drug release and hydrogels degradation *in vitro*

The cumulative release of Que in the Que-SLN@HA-PCLA group started to significantly differ from that of the Que@HA-PCLA group after 4 days (Fig. 3D). This difference increased over time, and the Que drug release rate in the Que-SLN@HA-PCLA group was ∼70% of the Que@HA-PCLA group after 21 days, with its release trend leveling off. Therefore, the introduction of SLN carriers enabled the sustained release of the Que hydrogel. This prevented the toxic effects of the rapid release of drugs in the bone repair microenvironment of the body.

However, some discrepancies between the two groups emerged after 21 days of hydrogel degradation among the analyses regarding the residual mass after hydrogel degradation (Fig. 3E). This implied that the use of SLNs as carriers facilitates Que hydrogel scaffold biodegradability to some extent.

### Biocompatibility of Que-SLN@HA-PCLA

**Figure 4.**
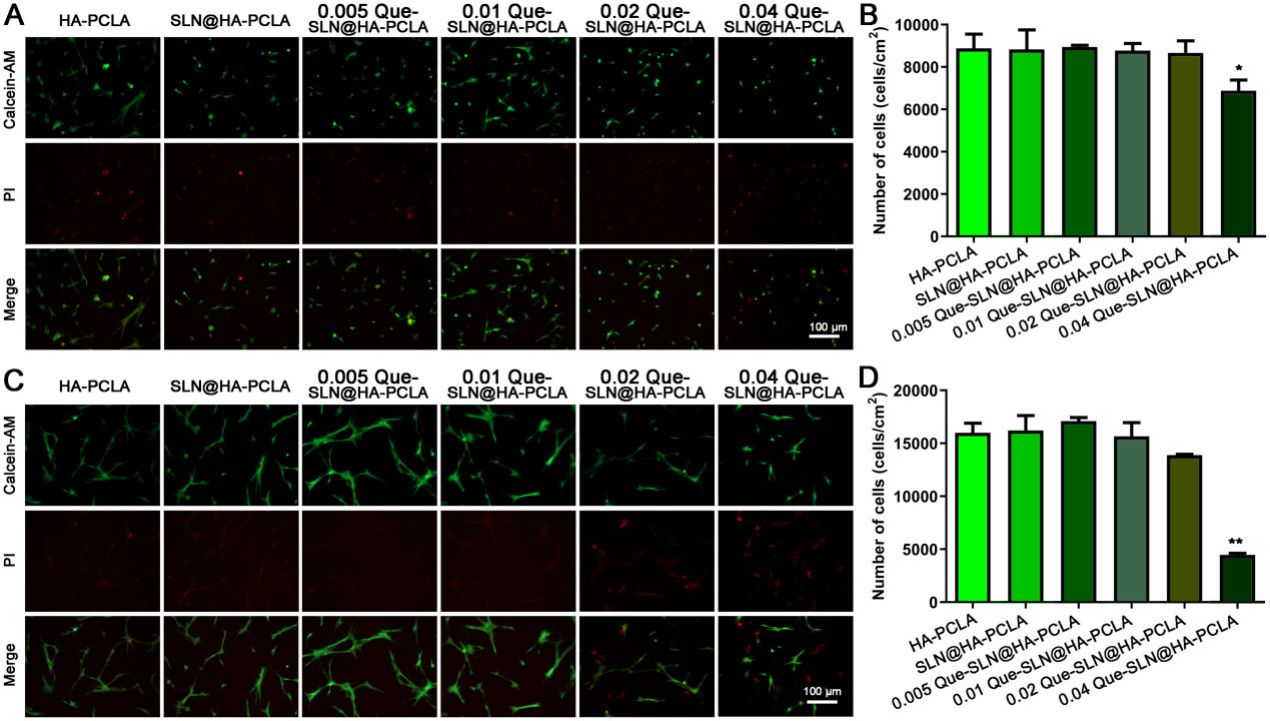
Cytocompatibility evaluation *in vitro*. (**A**, **B**) Fluorescence microscopy image and quantitative analysis of live dead cells of BMSCs cultured with different concentrations of Que-SLN@HA-PCLA hydrogels for 1 day, respectively. (**C**) Fluorescence image and (**D**) quantitative analysis of live dead cells of BMSCs cultured with different concentrations of Que-SLN@HA-PCLA hydrogels for 3 days. *P <* *0.05; *n* ≥ 3 for each group.

Cell viability was examined by live and dead cell staining (calcein-AM/PI double staining) to evaluate the biocompatibility of Que hydrogel composite scaffolds at different Que-SLN concentrations. Statistical analysis was performed after the implantation of the cells into the scaffolds on Days 1 and 3. Viable cells were labeled with green fluorescence by calcein-AM, while deceased cells were labeled with red fluorescence by PI (Fig. 4A and C). The number of live and dead cells in the HA-PCLA group was similar to that of the SLN@HA-PCLA group on Days 1 and 3 (Fig. 4B and D). This indicated that the addition of SLNs had little to no effect on BMSC activity.

Day 1 staining measurements showed that all groups had satisfactory cell survival and protrusion at both ends with the typical elongated shuttle-shaped morphology of BMSCs when the Que-SLN mass fraction in the hydrogel scaffold was below 0.01 (Fig. 4A and B). In contrast, the number of red-labeled dead cells was significantly higher when the mass fraction of Que-SLNs in hydrogel scaffolds reached 0.04. This indicated that the survival of BMSCs was suppressed. Staining results of Day 3 showed that the differentiation pattern of the 0.005 Que-SLN@HA-PCLA and 0.01 Que-SLN@HA-PCLA hydrogel holders were more pronounced, with a distinctly long shuttle shape and more robust and luxuriant protrusions at both ends (Fig. 4C). A longitudinal comparison of cell survival on Days 1 and 3 demonstrated that a greater number of cells survived over time in this composite hydrogel scaffold with a mass fraction of Que-SLNs above 0.02 compared to the pure hydrogel group without Que-SLNs (Fig. 4B and D).

A longitudinal comparison of cell survival demonstrates definite toxic effects in the group with a mass fraction of 0.04 Que-SLNs: the number of viable cells in the hydrogel decreased from over 6000 cells/cm^2^ on Day 1 to below 5000 cells/cm^2^ on Day 3. In contrast, the total number of viable cells slightly increased in the other groups. The 0.005 Que-SLN@HA-PCLA and 0.01 Que-SLN@HA-PCLA hydrogel scaffolds possess favorable cytocompatibility since they showed good proliferation of viable cells and better differentiation patterns compared to the other groups.

### Characterization of inflammatory modulation by Que-SLN@HA-PCLA

**Figure 5.**
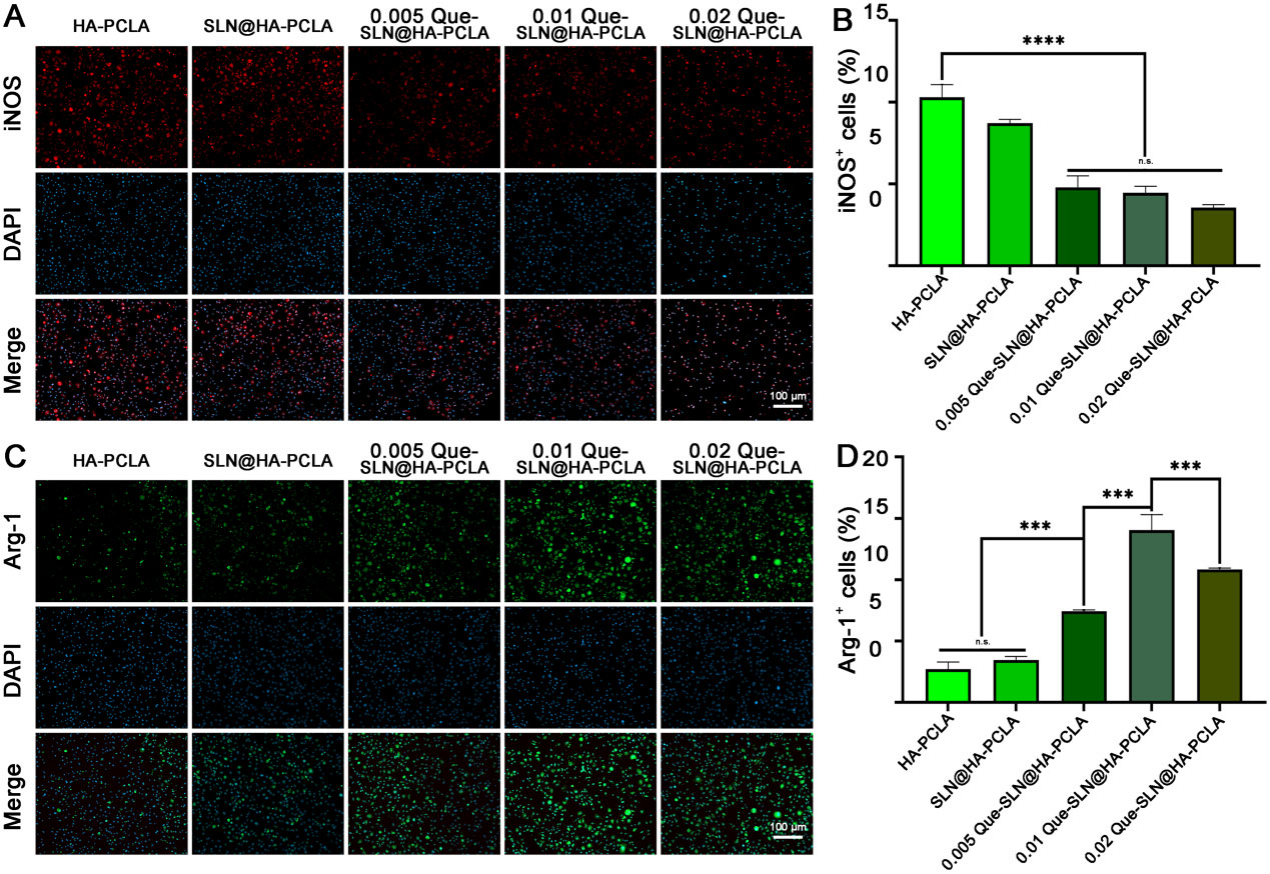
Immunoregulatory mechanism of hydrogel *in vitro*. (**A**, **B**) Fluorescence images and quantitative analysis results were obtained by immunofluorescence staining of cultured BMSCs with different Que-SLN@HA-PCLA concentrations, respectively. (**C**, **D**) Fluorescence images and quantitative analysis results were obtained by M2 macrophages, respectively. ***P <* *0.001 and ****P <* *0.0001; *n* ≥ 3 for each group; n.s. indicates no statistically different between the two groups.

The effect of different concentrations of Que scaffolds on the polarization of RAW264.7 macrophages was investigated by immunofluorescence. Cells were implanted into the different scaffolds for 2 days, followed by immunofluorescence staining with iNOS (M1 macrophage marker, red) and Arg-1 (M2 macrophage marker, green) primary antibodies. The fluorescence electron microscopy images (Fig. 5A) and the statistical data (Fig. 5B) showed that iNOS expression was low with the addition of Que, and the iNOS expression level gradually decreased with increasing Que concentration. The level of M2 polarization significantly increased after Que addition (Fig. 5C and D). The peak level of Arg-1 expression was reached when the mass fraction of Que-SLNs was 0.01, implying that this scaffold has the greatest ability to promote M2 polarization which correlates with the trend of osteogenesis-related detection.

### Osteogenesis of Que-SLN@HA-PCLA *in vitro*

**Figure 6.**
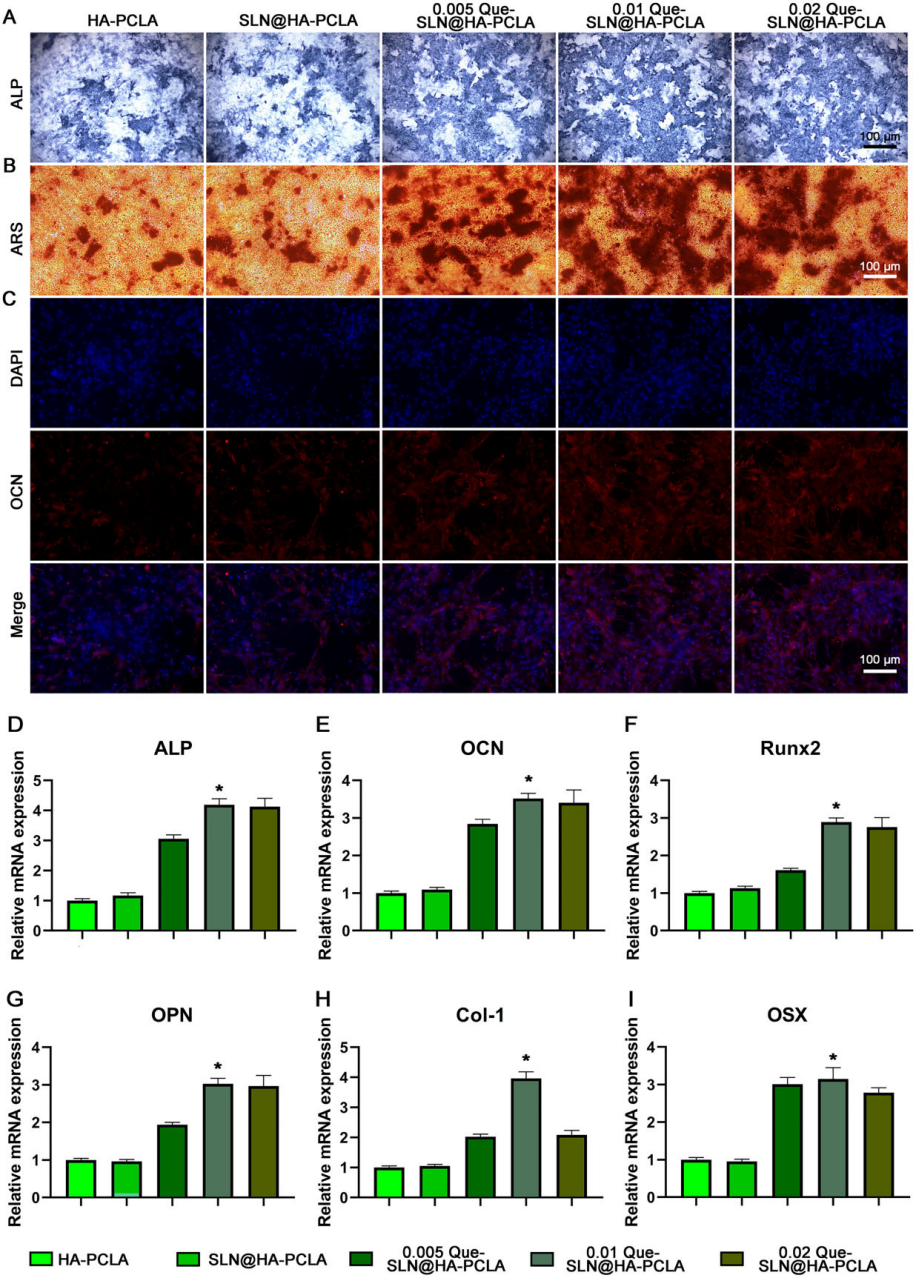
Evaluation of proliferation and osteogenic properties *in vitro*. (**A**) ALP staining of BMSCs cultured on different concentrations of Que-SLNs@HA-PCLA. The blue staining area represents ALP-positive cells. (**B**) ARS: the dark red staining area indicates generated bone nodules. (**C**) OCN immunofluorescence staining: red fluorescence represents OCN-positive cells, and blue fluorescence represents DAPI-labeled nuclei. RT-qPCR analysis of (**D**–**I**) ALP, OCN, Runx2, OPN, col-1 and OSX in BMSCs cultured on different concentrations of Que-SLN@HA-PCLA, respectively. *P <* *0.05; *n* ≥ 3 for each group.

The possible promotion of osteogenic differentiation by Que-SLN@HA-PCLA scaffolds was investigated by assessing ALP activity, calcium nodule content and OCN levels in BMSCs. ALP is a marker of early osteogenic differentiation of cells. No significant difference was seen between the HA-PCLA and SLN@HA-PCLA groups (Fig. 6A). This suggested that the addition of SLNs does not affect early osteogenic differentiation. ALP activity was higher in all scaffolds with Que addition than in the non-additional group, these results suggest the promotion of early osteogenic differentiation by Que-SLN@HA-PCLA scaffolds. ARS of mineralized nodules displayed a trend similar to that of ALP staining (Fig. 6B). The heavily stained red mineralized nodules in the 0.01 Que-SLN@HA-PCLA group were the most widely distributed among all scaffolds. Subsequently, immunofluorescence staining of OCN (a classical marker of late osteogenesis) revealed that the fraction of staining significantly increased when SLNs were used as the carrier (Fig. 6C). Similarly, the fluorescence staining area of OCN was most obvious in the 0.01 Que-SLN@HA-PCLA group. This suggested that the addition of SLNs loaded with Que to the HA-PCLA hydrogel scaffold enhanced late osteogenic differentiation, and this change was particularly evident in the 0.01 Que-SLN@HA-PCLA scaffold group.

RT-qPCR was performed to quantify the expression of osteogenic genes (including Alp, OCN, Runx2, OPN, Col-1 and OSX; Fig. 6D–I, respectively) and to investigate the effect of Que-SLNs@HA-PCLA on osteogenic differentiation. ALP expression in the HA-PCLA group was similar to that of the SLN@HA-PCLA groups. This indicated that the addition of SLNs did not affect gene expression. In contrast, ALP expression was higher in all scaffolds with added Que compared with the group lacking Que, especially in the 0.01 Que-SLN@HA-PCLA group. Similar results were observed in the expression of OCN, Runx2, OPN, Col-1 and OSX. This demonstrated that the Que-SLN@HA-PCLA scaffold promotes the expression of osteogenic genes when the Que-SLNs are loaded in HA-PCLA hydrogels at an optimal mass fraction of 0.01. Accordingly, the 0.01 Que-SLN@HA-PCLA scaffold was used in subsequent animal experiments.

### *In vivo* performance evaluation

#### Evaluation of bone reconstitution by imaging

**Figure 7.**
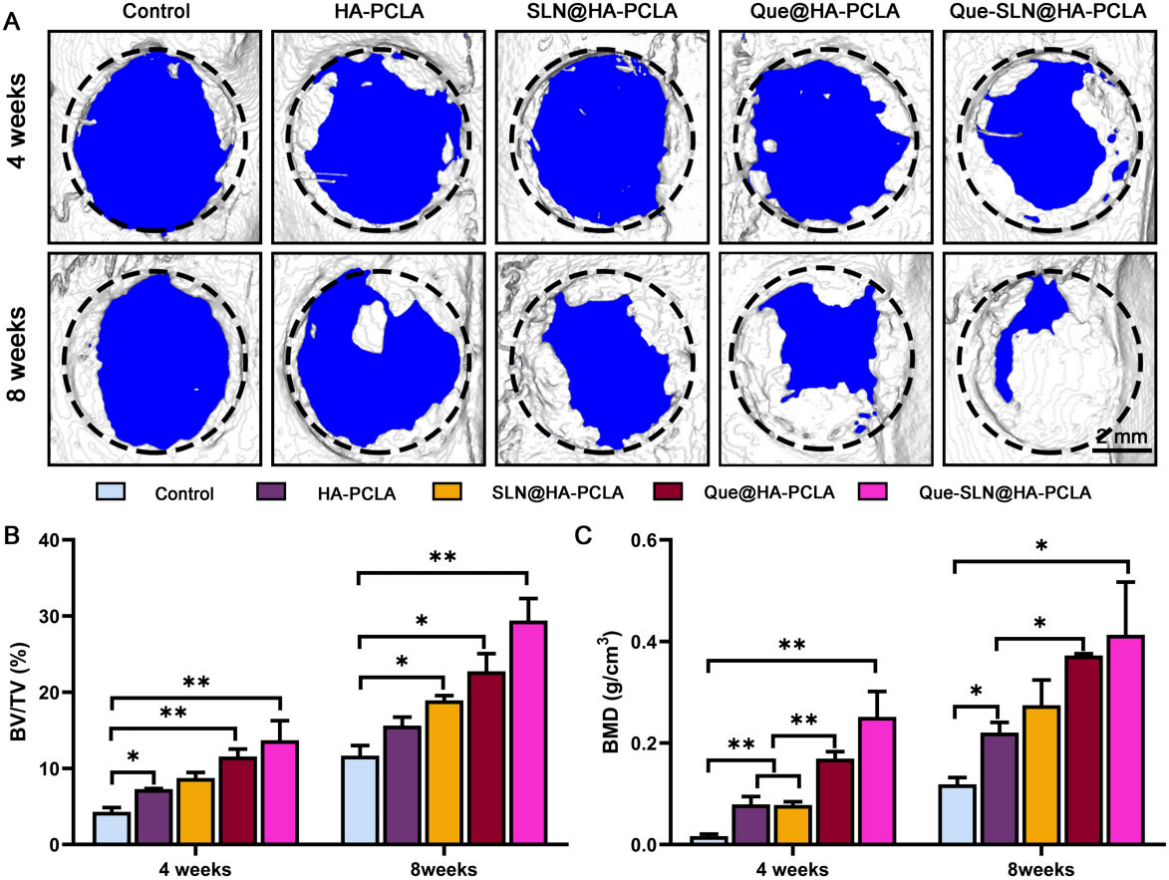
Micro-CT Analysis. (**A**) Representative 3D reconstructed image of skull defect after micro-CT scan. (**B**, **C**) BV/TV and BMD in the defect area, respectively. *P <* *0.05 and **P <* *0.01; *n* ≥ 3 for each group.

A critical-size cranial defect model was established in rats to evaluate the bone-healing effect of the scaffold. Rats undergoing surgery were well-conditioned and showed no signs of wound infection. Rats were postoperatively sacrificed at 4 and 8 weeks, and cranial bones were collected for micro-CT examination. No significant regenerative mineralized bone was observed at 4 weeks postoperation except for the two groups containing Que (Fig. 7A); the Que-SLN@HA-PCLA groups had slightly more regenerative mineralized bone than the Que@HA-PCLA group. The control group only had a small amount of new bone formation at the edges of the defect at 8 weeks postoperation, suggesting that it was difficult for the defect to spontaneously heal, which was consistent with the expected results. The regenerated bone in the HA-PCLA group and the SLN@HA-PCLA group showed a slight shift toward the center with a longer implantation time. New bone formation in the Que@HA-PCLA group and the Que-SLN@HA-PCLA group significantly increased compared to that in the other groups. In particular, the Que-SLN@HA-PCLA group showed new bone tissue filling almost the entire defect area at 8 weeks. The Que@HA-PCLA group and Que-SLN@HA-PCLA group reached over 30% BV/TV and had a BMD close to 0.4 at 8 weeks; this greatly exceeded those of the other groups. The Que-SLN@HA-PCLA groups promoted new bone formation to a significantly greater extent than the Que@HA-PCLA group when compared with the control group. This indicated that the SLN scaffold in these groups had the optimal effect in promoting new bone regeneration.

#### Histological evaluation of osteogenesis and osteoclasis *in vivo*

**Figure 8.**
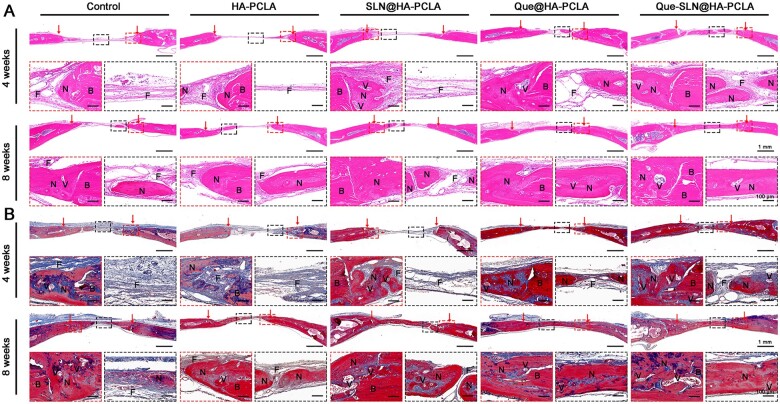
Representative images of skull defects using (**A**) H&E staining and (**B**) Masson’s trichromatic staining. H, N, V and F represent host bone, new bone, vessels and fibrous tissue, respectively.

The collected skull samples were decalcified and prepared into paraffin sections after micro-CT evaluation and metrological analysis. The scaffolds were completely degraded in all groups 4 weeks after the implantation according to H&E staining (Fig. 8A). The bone tissue stained a vibrant pink color, whereas the fibrous tissue was sparser and lighter in staining. In comparison, the control group, the HA-PCLA group, and the SLN@HA-PCLA groups showed defect areas mainly filled by fibrous tissue, with sparse new bone tissue only visible at the edges of the defects, and no significant growth of new bone tissue in the defect areas until 8 weeks after scaffold implantation. However, significant bone regeneration was observed in the Que@HA-PCLA group and the Que-SLN@HA-PCLA group, which had grown to the central area of the defect at 4 weeks and completely closed the defect area by 8 weeks after implantation. In particular, the thickness of the Que-SLN@HA-PCLA groups approached that of the host bone tissue, which correlated with the micro-CT analysis results. Masson’s staining showed dense blue- and red-stained osteoid islands in the bone tissue of the Que@HA-PCLA group and the Que-SLN@HA-PCLA groups (Fig. 8B). Furthermore, a few blood vessels were visible, which confirmed the significant promoting effect of Que-SLN@HA-PCLA on bone regenerative capacity.

**Figure 9.**
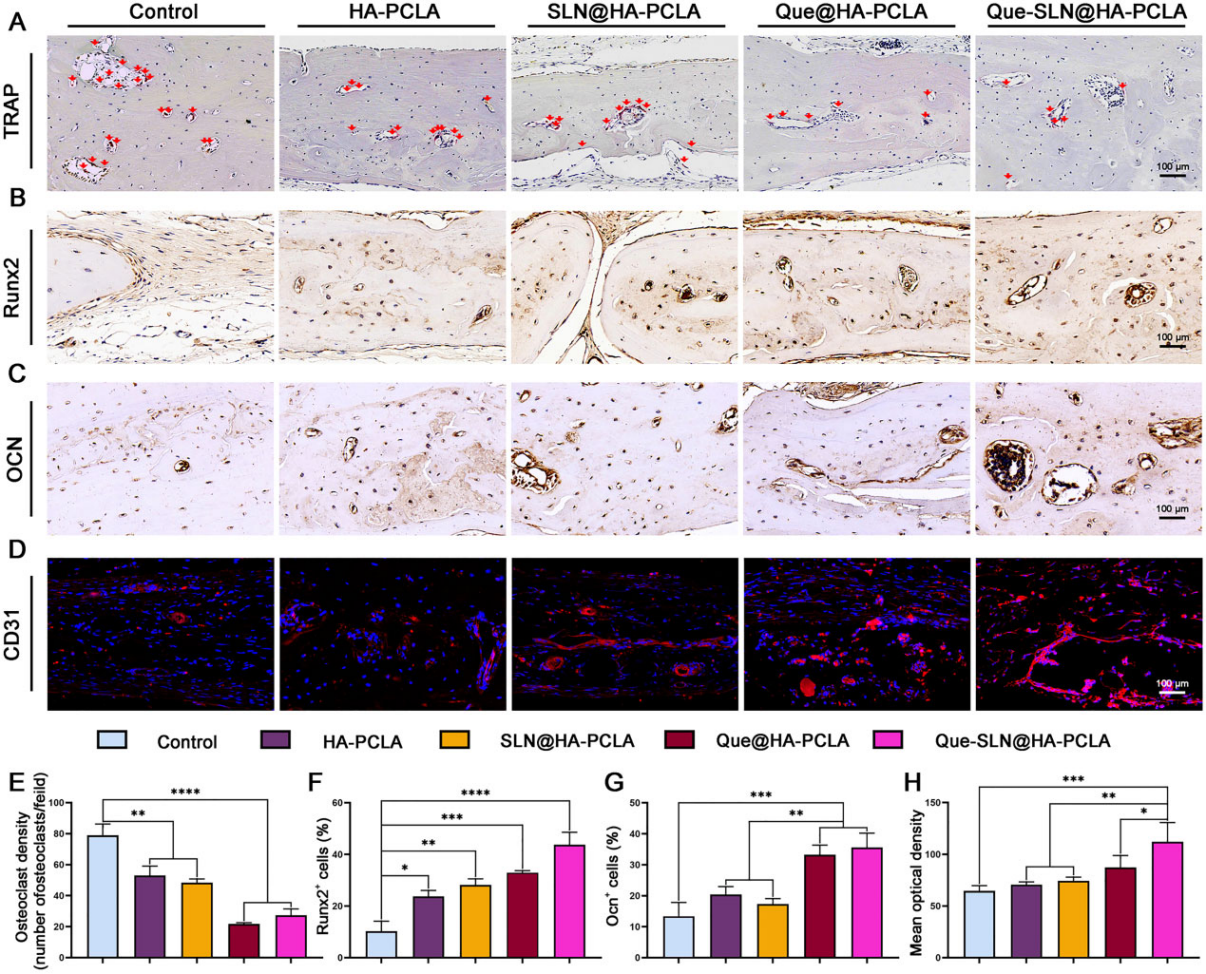
*In vivo* evaluation of osteoclastic, osteogenic, and vasculogenic abilities of hydrogel scaffolds. (**A**) TRAP staining with the dark blue-purple stained area indicated by the red arrow. (**B**) Runx2 immunohistochemical staining with dark brown areas. (**C**) OCN immunohistochemical staining with dark brown areas. (**D**) CD31 immunofluorescence staining with red fluorescence area. (**E**–**H**) TRAP staining, Runx2 immunohistochemical staining, OCN immunohistochemical staining and CD31 immunofluorescence staining were used for quantitative analysis. *P <* *0.05, **P <* *0.01, ***P <* *0.001 and ****P < 0.0001; *n* ≥ 3 for each group.

TRAP staining (Fig. 9A) is often used to show osteoclast activity, which is represented by wine-red-stained areas in the image. Osteoclast functionalization plays an important role in the bone repair process and is an indicator of bone reconstruction. Generally, osteoclast activity increases during active bone reconstruction. However, osteoclast activity was significantly reduced in the original PCLA hydrogel scaffold group compared to the control group. This reduction was amplified with the composite scaffold group loaded with Que to less than double that of the control group. Quantitative analysis of osteoclast numbers confirmed the histological observations. These results suggest that the Que with the PCLA hydrogel drives osteogenesis and reduces bone loss by inhibiting osteoclast activity (Fig. 9E). This extent of bone regeneration and mineralization is particularly pronounced after Que use and may be related to its inhibition of macrophage M1-type polarization.

In addition, immunohistochemical staining was performed using Runx2 and OCN as markers of osteogenic differentiation 8 weeks after implantation to investigate possible osteogenic-related expression during bone regeneration (Fig. 9B and C). These results combined with the immunohistochemical results (Fig. 9F and G) showed that the bone-related expression of Runx2 and OCN was higher in the two groups loaded with Que (especially in the Que-SLN@HA-PCLA groups) than that of the other groups. This correlated with the imaging and histological findings and indicated that Que-SLNs@HA-PCLA significantly accelerated matrix mineralization in the defect area and promoted bone reconstruction.

#### Histological evaluation of angiogenesis *in vivo*

Vascularization is another important indicator of bone regeneration, and the growth of new bone tissue is limited by its internal blood supply and the number of new capillaries surrounding it. The ability of different scaffold materials to mediate capillary angiogenesis *in vivo* was evaluated by performing and analyzing positive CD31 immunofluorescence staining in rat cranial sections after 4 weeks (Fig. 9D). The groups loaded with Que (especially the Que-SLN@HA-PCLA groups), had significantly better vascularization formation ability than that of the other groups (Fig. 9H).

### *In vivo* evaluation of the immunomodulatory effect

**Figure 10.**
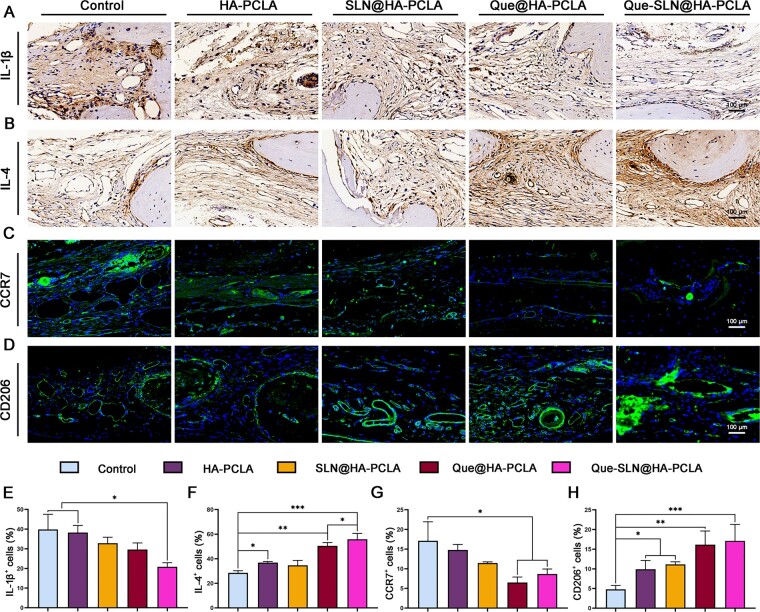
Evaluation of the immunoregulatory performance of hydrogel scaffolds *in vivo*. Immunohistochemical staining of the IL-1β (**A**) and IL-4 with dark brown areas. Immunofluorescence staining of CCR7 (**C**) and CD206 (**D**) with green fluorescence. (**E**–**H**) Quantitative analysis of IL-1β-, IL-4-, CCR7- and CD206-immunofluorescence staining. *P <* *0.05, **P <* *0.01 and ***P <* *0.001; *n* ≥ 3 for each group.

Macrophage polarization was examined in rat cranial sections after 4 weeks by using immunofluorescence staining to investigate the potential reasons for a series of improvements in bone regeneration in rats with skull defects (Fig. 10A). CCR7 (green fluorescence) is a relevant marker for M1-type macrophages and CD206 (green fluorescence) is a relevant marker for M2-type macrophages. More cells in the control group showed positive staining for CCR7 (Fig. 10E and H). Subsequent addition of the hydrogel scaffold (especially the group loaded with Que) showed lower levels of positive staining for CCR7. Meanwhile, the HA-PCLA group showed significantly higher CD206 expression levels than that of the control group, and this trend was amplified after loading with Que (especially in the Que-SLN@HA-PCLA groups) (Fig. 10B and F).

Expression of pro-inflammatory cytokine IL-1β significantly decreased in the group with the addition of hydrogel scaffold in cells in the skull defect area (especially in the Que-SLN@HA-PCLA groups compared with the control group) (Fig. 10C). In contrast, the expression of the anti-inflammatory cytokine, IL-4 (Fig. 10D and H), increased step-wise from the control group to the PCLA scaffold group to the Que@HA-PCLA scaffold group and the Que-SLN@HA-PCLA scaffold group.

## Discussion

Recent advances in tissue engineering approach combined with the induction of macrophage polarization to drive bone reconstruction at the defect site is encouraging. The incorporation of biomaterials with anti-inflammatory effects into the defective wound surface promotes the polarization of macrophages to the M2 type during inflammation due to contact or foreign body reaction [18]. The multiple regulatory molecules and anti-inflammatory factors derived from the polarization process are critical enablers for directing a series of bone tissue reconstructions. They promote the recruitment and differentiation of BMSCs, stimulate angiogenesis, regulate osteoclast homeostasis and enhance traditional tissue engineering therapies to amplify the effects of bone healing [21, 22]. This helps address the challenges of bone defect reconstruction. In this study, a hydrogel scaffold was designed and tested in a large-scale bone defect reconstruction study. It was based on the biological properties of Que to drive M2 polarization and contains a hydrogel structure built with an HA component exhibiting immunomodulatory functions. This is a potential new strategy for driving bone reconstruction.

The immunomodulatory properties of hydrogels are usually slightly influenced by their construction and properties [58]. The surface of the HA-PCLA material was flat and smooth according to the SEM results. In comparison, the surface morphology of the SLNs embedded in the hydrogel was rougher with a micro-crest shape. The attachment of macrophages is significantly enhanced on the surfaces of biomaterials with distinct morphologies compared to flat surfaces [23, 59]. Therefore, the change in the surface morphology of the hydrogel caused by the loading of SLNs increased the surface contact area between the material and the defective wound (providing more areas for cell adhesion) and enhanced the adhesion of the material surface to macrophages to facilitate their immunomodulation. Que is a natural plant extract with low toxicity and side effects and anti-inflammatory effects. However, the sudden release of large amounts of drugs can aggravate the foreign body reaction, lead to unnecessary inflammatory processes and enhance the release of inflammatory factors. This is detrimental to the immune regulation of the osteogenic microenvironment [60]. In contrast, slow Que release can promote full contact of the Que with the wound and better exert its immunomodulatory effect on macrophages. Meanwhile, the Que-SLN@HA-PCLA scaffolds showed a higher mass loss in PBS compared with the Que@HA-PCLA group. This indicated that their biodegradability *in vivo* was enhanced, which facilitated the penetration of the HA component of the hydrogel into the tissue. The acidic functional groups in the HA component are beneficial to further enhance the scaffold's capacity to elicit *in situ* biomineralization [45].

Analysis of cell viability by live-dead cell staining assay showed that SLN embedding in hydrogels did not affect BMSC activity. However, the cell growth of BMSCs was inhibited when the dose of Que was too high and its mass fraction was over 0.04. This was more significant at 3 days versus 1 day after the transplant. In contrast, cell viability was slightly better than the other doses of the group at a Que mass fraction of 0.005–0.01 in the scaffold, and it was somewhat enhanced compared to the control. The cytocompatibility of Que-SLN@HA-PCLA scaffolds was dose-dependent. By detecting the effect of different concentrations of Que scaffolds on macrophage polarization by immunofluorescence, we confirmed that the addition of Que inhibited M1-type polarization levels and enhanced M2-type polarization levels. We hypothesized that Que exerts its powerful immunomodulatory effects by regulating macrophage polarization, thereby promoting a more favorable immune microenvironment for osteogenesis in the vicinity of the biologic scaffold material. In subsequent assays of scaffold osteogenic capacity, the advantage of the 0.01 Que-SLN@HA-PCLA scaffold was particularly evident in the fluorescent staining of ALP, ARS and OCN osteogenic capacity-related expression. This may be due to the stronger cell viability of the scaffold in the corresponding groups. However, RT-qPCR results showed a significant difference in osteogenic capacity-related expression in the 0.01 Que-SLN@HA-PCLA scaffold compared with the other groups, especially compared with the pure HA-PCLA group without Que addition versus the SLN@HA-PCLA scaffold group. This result was inconsistent with the results of the cell viability assay. In contrast, there was no difference in the expression of bone-related genes in the pure HA-PCLA group versus the SLN@HA-PCLA group without Que addition. This indicates that this change mainly originated from Que addition. Moreover, the trend of osteogenic enhancement exhibited by their group comparisons remained consistent with the previously measured M2 polarization levels, with the 0.01 Que-SLN@HA-PCLA scaffold exhibiting the strongest M2-type polarization levels. This also confirms our previous hypothesis indicating that *in vitro* Que immunomodulates by regulating the polarization of macrophages to promote the reconstruction of defective bone. This makes the immune microenvironment more favorable for osteogenesis.

CT images in the critical-sized rat bone defect model, combined with the results of BV/TV and BMD quantification in each scaffold group, showed a gradient increase in BV and bone density when HA-PCLA, SLN@HA-PCLA, Que@HA-PCLA and Que-SLN@HA-PCLA were added, respectively. The trend of this gradient became more pronounced over time. Similar trends were observed with Masson’s trichrome staining and H&E staining, with a more pronounced grouping of firm new bone and a clear expansion in the mature bone region. The increased osteogenesis, due to the loading of SLNs, can be attributed to the enhanced macrophage attachment capacity due to its alteration of the hydrogel surface morphology to a rough microcrest. Similarly, we hypothesize that one of the most obvious changes (the significant increase in osteogenesis caused by Que loading) is mainly due to the increase in macrophage-derived molecular factors associated with bone regeneration due to the formation of an anti-inflammatory microenvironment caused by the increased M2-type polarization of macrophages under the influence of Que.

Mechanisms by which biomaterials promote bone regeneration include immunomodulation by macrophages. This is crucial for the interaction between implant materials and the microenvironment at the bone defect. Increasing evidence suggests that the regulation of macrophages by Que is derived from the inhibition of the NF-κB pathway [32–34]. It significantly reduces the levels of macrophage M1 polarization markers IL-1β, IL-6 and TNF-α, while activating the AMPK and Akt signaling pathways [32, 33] to enhance M2 polarization levels and increase the expression of anti-inflammatory factors. Quantitative analysis showed that IL-1β expression significantly decreased while IL-4 expression increased with the introduction of hydrogel, vector and Que (especially in the Que-SLN@HA-PCLA groups). This proves that the introduction of Que exerts a significant function in the regulation of the osteogenic microenvironment in bone defects. Immunofluorescence staining of M1 and M2 specifically expressed proteins CCR7 and CD206, which further revealed the intrinsic regulatory mechanism of the Que scaffold on macrophage polarization. The implantation of different composite hydrogel scaffolds suppressed M1-type polarization, while M2-type polarization significantly increased.

Meanwhile, immunohistochemical staining for OCN and RunX2, and fluorescent staining for CD31 showed a similar trend of increase for Que-SLN@HA-PCLA to that of all other groups. M2 macrophages enhance bone regeneration by secreting the BMP-2 with VEGF signaling, which promotes angiogenesis and induces bone differentiation [17, 19]. Our results suggest that the Que composite scaffold enhances BMP-2 and VEGF paracrine signaling in M2 macrophages to trigger osteoblast differentiation and vascularization in BMSCs, with the synergistic enhancement of bone regeneration. Polarization of M2-type macrophages plays a significant role in immunomodulation to obtain an anti-inflammatory immune microenvironment conducive to bone regeneration. It facilitates the coordination of angiogenesis and osteogenesis during bone repair and further enhances bone regeneration. Quantitative analysis by TRAP staining showed that Que significantly inhibited the osteolytic process and promoted the osteogenic-osteolytic balance toward osteogenesis.

The Que composite scaffold (Que-SLN@HA-PCLA) induced M2 polarization to modulate the local immune microenvironment to promote bone regeneration, where the polarization effect of driving macrophages is dominated by Que release. This is crucial for the Que composite scaffold to drive bone regeneration. The surface conformation of the hydrogel scaffold and the anti-inflammatory component of HA in the hydrogel play an assistive role. In conclusion, the Que composite scaffold provides a new approach to the development of immunomodulatory biomaterials to promote the regeneration of defective bone. However, the mechanism of macrophage recruitment by Que release is unknown. Further studies are required to determine whether Que release enhances the release or effect of key chemokines of macrophages. Additionally, it is unclear whether the chemical composition of the lipid carrier particle affects the immunomodulatory program.

## Conclusion

We proposed a method to promote bone regeneration in large-scale bone defects through Que-SLN@HA-PCLA composite hydrogel implantation that focuses on the immunomodulation of macrophages. A series of cellular experiments demonstrated that the composite hydrogel inhibited the polarization of M1-type macrophages and enhanced the polarization of M2 to promote osteogenesis *in vitro*. We further demonstrated modification of the immune microenvironment in the defect area by using a rat model of severe cranial defects and detected a significant enhancement of bone regeneration and angiogenesis with an elevated level of M2 polarization. In conclusion, this composite scaffold provides a new approach for utilizing biomaterials to promote defective bone regeneration through immunomodulation and has great potential for application in the regenerative treatment of large-scale bone defects. However, there is a lack of validation in larger animals, and the specific mechanism of Que affecting macrophage polarization remains unclear, thus its clinical application is still to be evaluated in further experimental studies.

## Supplementary Material

rbad025_Supplementary_Data

## References

[1] Wojtowicz AM , Shekaran A, Oest ME, Dupont KM, Templeman KL, Hutmacher DW, Guldberg RE, Garcia AJ. Coating of biomaterial scaffolds with the collagen-mimetic peptide GFOGER for bone defect repair. Biomaterials 2010;31:2574–82. 20056517 10.1016/j.biomaterials.2009.12.008 PMC2813962

[2] Collon K , Gallo MC, Lieberman JR. Musculoskeletal tissue engineering: regional gene therapy for bone repair. Biomaterials 2021;275:120901. 34091300 10.1016/j.biomaterials.2021.120901

[3] Mao Y , Chen Y, Li W, Wang Y, Qiu J, Fu Y, Guan J, Zhou P. Physiology-inspired multilayer nanofibrous membranes modulating endogenous stem cell recruitment and osteo-differentiation for staged bone regeneration. Adv Healthc Mater 2022;11:e2201457. 36027596 10.1002/adhm.202201457

[4] Wubneh A , Tsekoura EK, Ayranci C, Uludag H. Current state of fabrication technologies and materials for bone tissue engineering. Acta Biomater 2018;80:1–30. 30248515 10.1016/j.actbio.2018.09.031

[5] Shao N , Guo J, Guan Y, Zhang H, Li X, Chen X, Zhou D, Huang Y. Development of organic/inorganic compatible and sustainably bioactive composites for effective bone regeneration. Biomacromolecules 2018;19:3637–48. 30049206 10.1021/acs.biomac.8b00707

[6] Wu J , Yao M, Zhang Y, Lin Z, Zou W, Li J, Habibovic P, Du C. Biomimetic three-layered membranes comprising (poly)-epsilon-caprolactone, collagen and mineralized collagen for guided bone regeneration. Regen Biomater 2021;8:rbab065. 34881047 10.1093/rb/rbab065 PMC8648192

[7] He W , Bai J, Chen X, Suo D, Wang S, Guo Q, Yin W, Geng D, Wang M, Pan G, Zhao X, Li B. Reversible dougong structured receptor-ligand recognition for building dynamic extracellular matrix mimics. Proc Natl Acad Sci USA 2022;119:e2117221119. 35181608 10.1073/pnas.2117221119 PMC8872741

[8] Li W , Yang X, Lai P, Shang L. Bio-inspired adhesive hydrogel for biomedicine—principles and design strategies. Smart Med 2022;1:e20220024. 10.1002/SMMD.20220024 PMC11235927 39188733

[9] Lei Y , Zhang Q, Kuang G, Wang X, Fan Q, Ye F. Functional biomaterials for osteoarthritis treatment: from research to application. Smart Med 2022;1:e20220014. 10.1002/SMMD.20220014 PMC11235767 39188730

[10] Wang Y , Wang J, Gao R, Liu X, Feng Z, Zhang C, Huang P, Dong A, Kong D, Wang W. Biomimetic glycopeptide hydrogel coated PCL/nHA scaffold for enhanced cranial bone regeneration via macrophage M2 polarization-induced osteo-immunomodulation. Biomaterials 2022;285:121538. 35504180 10.1016/j.biomaterials.2022.121538

[11] Zhang Y , Li Z, Wang Z, Yan B, Shi A, Xu J, Guan J, Zhang L, Zhou P, Mao Y. Mechanically enhanced composite hydrogel scaffold for in situ bone repairs. Biomater Adv 2022;134:112700. 35581085 10.1016/j.msec.2022.112700

[12] Zhang W , Dai X, Jin X, Huang M, Shan J, Chen X, Qian H, Chen Z, Wang X. Promotion of wound healing by a thermosensitive and sprayable hydrogel with nanozyme activity and anti-inflammatory properties. Smart Mater Med 2023;4:134–45.

[13] Dewey MJ , Johnson EM, Slater ST, Milner DJ, Wheeler MB, Harley BAC. Mineralized collagen scaffolds fabricated with amniotic membrane matrix increase osteogenesis under inflammatory conditions. Regen Biomater 2020;7:247–58. 32523727 10.1093/rb/rbaa005 PMC7266662

[14] Zhang J , Tong D, Song H, Ruan R, Sun Y, Lin Y, Wang J, Hou L, Dai J, Ding J, Yang H. Osteoimmunity-regulating biomimetically hierarchical scaffold for augmented bone regeneration. Adv Mater 2022;34:e2202044. 35785450 10.1002/adma.202202044

[15] Qiu P , Li M, Chen K, Fang B, Chen P, Tang Z, Lin X, Fan S. Periosteal matrix-derived hydrogel promotes bone repair through an early immune regulation coupled with enhanced angio- and osteogenesis. Biomaterials 2020;227:119552. 31670079 10.1016/j.biomaterials.2019.119552

[16] Kim H , Joo Y, Kook YM, Tran NL, Kim SH, Lee K, Oh SJ. On-demand local immunomodulation via epigenetic control of macrophages using an inflammation-responsive hydrogel for accelerated wound healing. ACS Appl Mater Interfaces 2022;14:4931–45. 34989546 10.1021/acsami.1c20394

[17] Zhang J , Shi H, Zhang N, Hu L, Jing W, Pan J. Interleukin-4-loaded hydrogel scaffold regulates macrophages polarization to promote bone mesenchymal stem cells osteogenic differentiation via TGF-beta1/Smad pathway for repair of bone defect. Cell Prolif 2020;53:e12907. 32951298 10.1111/cpr.12907 PMC7574882

[18] Liang L , Song D, Wu K, Ouyang Z, Huang Q, Lei G, Zhou K, Xiao J, Wu H. Sequential activation of M1 and M2 phenotypes in macrophages by Mg degradation from Ti-Mg alloy for enhanced osteogenesis. Biomater Res 2022;26:17. 35484564 10.1186/s40824-022-00262-w PMC9052665

[19] Ji X , Shao H, Li X, Ullah MW, Luo G, Xu Z, Ma L, He X, Lei Z, Li Q, Jiang X, Yang G, Zhang Y. Injectable immunomodulation-based porous chitosan microspheres/HPCH hydrogel composites as a controlled drug delivery system for osteochondral regeneration. Biomaterials 2022;285:121530. 35504181 10.1016/j.biomaterials.2022.121530

[20] Sadowska JM , Wei F, Guo J, Guillem-Marti J, Ginebra MP, Xiao Y. Effect of nano-structural properties of biomimetic hydroxyapatite on osteoimmunomodulation. Biomaterials 2018;181:318–32. 30098568 10.1016/j.biomaterials.2018.07.058

[21] Tan S , Wang Y, Du Y, Xiao Y, Zhang S. Injectable bone cement with magnesium-containing microspheres enhances osteogenesis via anti-inflammatory immunoregulation. Bioact Mater 2021;6:3411–23. 33842737 10.1016/j.bioactmat.2021.03.006 PMC8010581

[22] Jiang G , Li S, Yu K, He B, Hong J, Xu T, Meng J, Ye C, Chen Y, Shi Z, Feng G, Chen W, Yan S, He Y, Yan R. A 3D-printed PRP-GelMA hydrogel promotes osteochondral regeneration through M2 macrophage polarization in a rabbit model. Acta Biomater 2021;128:150–62. 33894346 10.1016/j.actbio.2021.04.010

[23] Vassey MJ , Figueredo GP, Scurr DJ, Vasilevich AS, Vermeulen S, Carlier A, Luckett J, Beijer NRM, Williams P, Winkler DA, de Boer J, Ghaemmaghami AM, Alexander MR. Immune modulation by design: using topography to control human monocyte attachment and macrophage differentiation. Adv Sci (Weinh) 2020;7:1903392. 32537404 10.1002/advs.201903392 PMC7284204

[24] Liu X , Chen M, Luo J, Zhao H, Zhou X, Gu Q, Yang H, Zhu X, Cui W, Shi Q. Immunopolarization-regulated 3D printed-electrospun fibrous scaffolds for bone regeneration. Biomaterials 2021;276:121037. 34325336 10.1016/j.biomaterials.2021.121037

[25] Sun X , Ma Z, Zhao X, Zhang JW, Ma C, Qiang J, Wang L, Deng W, Yang Q, Zhao H, Liang J, Zhou Q, Li X, Wang T. J. Three-dimensional bioprinting of multicell-laden scaffolds containing bone morphogenic protein-4 for promoting M2 macrophage polarization and accelerating bone defect repair in diabetes mellitus. Bioact Mater 2021;6:757–69. 33024897 10.1016/j.bioactmat.2020.08.030 PMC7522044

[26] Pan X , Yuan S, Xun X, Fan Z, Xue X, Zhang C, Wang J, Deng J. Long-Term recruitment of endogenous M2 macrophages by platelet Lysate-Rich plasma macroporous hydrogel scaffold for articular cartilage defect repair. Adv Healthc Mater 2022;11:e2101661. 34969180 10.1002/adhm.202101661

[27] Wu M , Zhang Y, Wu P, Chen F, Yang Z, Zhang S, Xiao L, Cai L, Zhang C, Chen Y, Deng Z. Mussel-inspired multifunctional surface through promoting osteogenesis and inhibiting osteoclastogenesis to facilitate bone regeneration. NPJ Regen Med 2022;7:29. 35562356 10.1038/s41536-022-00224-9 PMC9106696

[28] Bai Z , Hu K, Shou Z, Yu J, Meng H, Zhou H, Chen L, Yu T, Lu R, Li N, Chen C. Layer-by-layer assembly of procyanidin and collagen promotes mesenchymal stem cell proliferation and osteogenic differentiation in vitro and in vivo. Regen Biomater 2023;10:rbac107. 36683760 10.1093/rb/rbac107 PMC9847536

[29] Wan J , Benkdane M, Teixeira-Clerc F, Bonnafous S, Louvet A, Lafdil F, Pecker F, Tran A, Gual P, Mallat A, Lotersztajn S, Pavoine C. M2 Kupffer cells promote M1 Kupffer cell apoptosis: a protective mechanism against alcoholic and nonalcoholic fatty liver disease. Hepatology 2014;59:130–42. 23832548 10.1002/hep.26607

[30] Subedi L , Gaire BP. Phytochemicals as regulators of microglia/macrophages activation in cerebral ischemia. Pharmacol Res 2021;165:105419. 33450385 10.1016/j.phrs.2021.105419

[31] Song JE , Tripathy N, Lee DH, Park JH, Khang G. Quercetin inlaid silk fibroin/hydroxyapatite scaffold promotes enhanced osteogenesis. ACS Appl Mater Interfaces 2018;10:32955–64. 30188112 10.1021/acsami.8b08119

[32] Lu H , Wu L, Liu L, Ruan Q, Zhang X, Hong W, Wu S, Bai JG. Y. Quercetin ameliorates kidney injury and fibrosis by modulating M1/M2 macrophage polarization. Biochem Pharmacol 2018;154:203–12. 29753749 10.1016/j.bcp.2018.05.007

[33] Wang Y , Li C, Wan Y, Qi M, Chen Q, Sun Y, Sun X, Fang J, Fu L, Xu L, Dong B, Wang L. Quercetin-loaded ceria nanocomposite potentiate dual-directional immunoregulation via macrophage polarization against periodontal inflammation. Small 2021;17:e2101505. 34499411 10.1002/smll.202101505

[34] Wong RW , Rabie AB. Effect of quercetin on bone formation. J Orthop Res 2008;26:1061–6. 18383168 10.1002/jor.20638

[35] Wang Y , Quan F, Cao Q, Lin Y, Yue C, Bi R, Cui X, Yang H, Yang Y, Birnbaumer L, Li X, Gao X. Quercetin alleviates acute kidney injury by inhibiting ferroptosis. J Adv Res 2021;28:231–43. 33364059 10.1016/j.jare.2020.07.007 PMC7753233

[36] Wong SK , Chin KY, Ima-Nirwana S. Quercetin as an agent for protecting the bone: a review of the current evidence. Int J Mol Sci 2020;21:6448. 32899435 10.3390/ijms21176448 PMC7503351

[37] Han X , Xu T, Fang Q, Zhang H, Yue L, Hu G, Sun L. Quercetin hinders microglial activation to alleviate neurotoxicity via the interplay between NLRP3 inflammasome and mitophagy. Redox Biol 2021;44:102010. 34082381 10.1016/j.redox.2021.102010 PMC8182123

[38] Salunkhe SA , Chitkara D, Mahato RI, Mittal A. Lipid based nanocarriers for effective drug delivery and treatment of diabetes associated liver fibrosis. Adv Drug Deliv Rev 2021;173:394–415. 33831474 10.1016/j.addr.2021.04.003

[39] McClements DJ. Advances in nanoparticle and microparticle delivery systems for increasing the dispersibility, stability, and bioactivity of phytochemicals. Biotechnol Adv 2020;38:107287. 30086329 10.1016/j.biotechadv.2018.08.004

[40] Gu Y , Yang M, Tang X, Wang T, Yang D, Zhai G, Liu J. Lipid nanoparticles loading triptolide for transdermal delivery: mechanisms of penetration enhancement and transport properties. J Nanobiotechnology 2018;16:68. 30217198 10.1186/s12951-018-0389-3 PMC6138933

[41] Nunes S , Madureira AR, Campos D, Sarmento B, Gomes AM, Pintado M, Reis F. Solid lipid nanoparticles as oral delivery systems of phenolic compounds: overcoming pharmacokinetic limitations for nutraceutical applications. Crit Rev Food Sci Nutr 2017;57:1863–73. 26192708 10.1080/10408398.2015.1031337

[42] Wu X , Chen H, Wu C, Wang J, Zhang S, Gao J, Wang H, Sun T, Yang YG. Inhibition of intrinsic coagulation improves safety and tumor-targeted drug delivery of cationic solid lipid nanoparticles. Biomaterials 2018;156:77–87. 29190500 10.1016/j.biomaterials.2017.11.040

[43] Permana AD , Tekko IA, McCrudden MTC, Anjani QK, Ramadon D, McCarthy HO, Donnelly RF. Solid lipid nanoparticle-based dissolving microneedles: a promising intradermal lymph targeting drug delivery system with potential for enhanced treatment of lymphatic filariasis. J Control Release 2019;316:34–52. 31655132 10.1016/j.jconrel.2019.10.004

[44] Wei B , Wang W, Liu X, Xu C, Wang Y, Wang Z, Xu J, Guan J, Zhou P, Mao Y. Gelatin methacrylate hydrogel scaffold carrying resveratrol-loaded solid lipid nanoparticles for enhancement of osteogenic differentiation of BMSCs and effective bone regeneration. Regen Biomater 2021;8:rbab044. 34394955 10.1093/rb/rbab044 PMC8358478

[45] Kim SH , Thambi T, Giang Phan VH, Lee DS. Modularly engineered alginate bioconjugate hydrogel as biocompatible injectable scaffold for in situ biomineralization. Carbohydr Polym 2020;233:115832. 32059885 10.1016/j.carbpol.2020.115832

[46] Duong HTT , Thambi T, Yin Y, Kim SH, Nguyen TL, Phan VHG, Kim J, Jeong JH, Lee DS. Degradation-regulated architecture of injectable smart hydrogels enhances humoral immune response and potentiates antitumor activity in human lung carcinoma. Biomaterials 2020;230:119599. 31718883 10.1016/j.biomaterials.2019.119599

[47] Guo J , Luo Z, Wang F, Gu H, Li M. Responsive hydrogel microfibers for biomedical engineering. Smart Medicine 2022;1:e20220003. 10.1002/SMMD.20220003 PMC11235791 39188750

[48] Zheng H , Wang N, Li L, Ge L, Jia H, Fan Z. miR-140-3p enhanced the osteo/odontogenic differentiation of DPSCs via inhibiting KMT5B under hypoxia condition. Int J Oral Sci 2021;13:41. 34876565 10.1038/s41368-021-00148-y PMC8651682

[49] Livak KJ , Schmittgen TD. Analysis of relative gene expression data using real-time quantitative PCR and the 2(-Delta Delta C(T)) method. Methods 2001;25:402–8. 11846609 10.1006/meth.2001.1262

[50] Wang X , Lin M, Kang Y. Engineering porous beta-tricalcium phosphate (beta-TCP) scaffolds with multiple channels to promote cell migration, proliferation, and angiogenesis. ACS Appl Mater Interfaces 2019;11:9223–32. 30758175 10.1021/acsami.8b22041

[51] Song JE , Tian J, Kook YJ, Thangavelu M, Choi JH, Khang G. A BMSCs-laden quercetin/duck's feet collagen/hydroxyapatite sponge for enhanced bone regeneration. J Biomed Mater Res A 2020;108:784–94. 31794132 10.1002/jbm.a.36857

[52] Jung JM , Kim SH, Giang Phan VH, Thambi T, Lee DS. Therapeutic effects of boronate ester cross-linked injectable hydrogels for the treatment of hepatocellular carcinoma. Biomater Sci 2021;9:7275–86. 34609388 10.1039/d1bm00881a

[53] Cui Y , Jin R, Zhang Y, Yu M, Zhou Y, Wang LQ. Cellulose nanocrystal-enhanced thermal-sensitive hydrogels of block copolymers for 3D bioprinting. Int J Bioprint 2021;7:397. 34805591 10.18063/ijb.v7i4.397 PMC8600300

[54] Zhang Z , Cui H. Biodegradability and biocompatibility study of poly(chitosan-g-lactic acid) scaffolds. Molecules 2012;17:3243–58. 22418927 10.3390/molecules17033243 PMC6268052

[55] Jin S , Pu Y, Guo Z, Zhu W, Li S, Zhou X, Gao W, He B. A double-layer dura mater based on poly(caprolactone-co-lactide) film and polyurethane sponge: preparation, characterization, and biodegradation study. J Mater Chem B 2021;9:3863–73. 33928320 10.1039/d1tb00454a

[56] Phan VHG , Murugesan M, Manivasagan P, Nguyen TL, Phan TH, Luu CH, Ho DK, Li Y, Kim J, Lee DS, Thambi T. Injectable hydrogel based on protein-polyester microporous network as an implantable niche for active cell recruitment. Pharmaceutics 2022;14:709. 35456546 10.3390/pharmaceutics14040709 PMC9024632

[57] Mohan R , Mohan N, Vaikkath D. Hyaluronic acid dictates chondrocyte morphology and migration in composite gels. Tissue Eng Part A 2018;24:1481–91. 29681215 10.1089/ten.TEA.2017.0411

[58] Kharaziha M , Baidya A, Annabi N. Rational design of immunomodulatory hydrogels for chronic wound healing. Adv Mater 2021;33:e2100176. 34251690 10.1002/adma.202100176 PMC8489436

[59] Singh S , Awuah D, Rostam HM, Emes RD, Kandola NK, Onion D, Htwe SS, Rajchagool B, Cha BH, Kim D, Tighe PJ, Vrana NE, Khademhosseini A, Ghaemmaghami A. Unbiased analysis of the impact of micropatterned biomaterials on macrophage behavior provides insights beyond predefined polarization states. ACS Biomater Sci Eng 2017;3:969–78. 33429569 10.1021/acsbiomaterials.7b00104

[60] Welch NG , Winkler DA, Thissen H. Antifibrotic strategies for medical devices. Adv Drug Deliv Rev 2020;167:109–20. 32553685 10.1016/j.addr.2020.06.008

