# Supplementary material for: Quercetin-solid lipid nanoparticle-embedded hyaluronic acid functionalized hydrogel for immunomodulation to promote bone reconstruction
Source: Regen Biomater. 2023 Apr 11;10:rbad025. doi: 10.1093/rb/rbad025 (PMC10110271; doi:10.1093/rb/rbad025)
Supplement: rbad025_Supplementary_Data [file rbad025_supplementary_data.docx]

**Quercetin-solid lipid nanoparticle-embedded hyaluronic acid functionalized hydrogel for immunomodulation to promote bone reconstruction**

Pinghui Zhou ^1,3,†^, Bomin Yan ^1,†^, Bangguo Wei ^1^, Liangmin Fu ^1^, Ying Wang ^1^, Wenrui Wang ^2,4,*^, Li Zhang ^1,3,*^, Yingji Mao ^1,2,3,*^

*^1^ Department of Orthopaedics and Department of Plastic surgery, The First Affiliated Hospital of Bengbu Medical College, Bengbu, 233004, China*

*^2^ School of Life Science, Bengbu Medical College, Bengbu 233030, China*

*^3^ Anhui Province Key Laboratory of Tissue Transplantation, Bengbu Medical College, Bengbu 233030, China*

*^4^ Anhui Province Key Laboratory of Translational Cancer Research, Bengbu Medical University, Anhui 233030, China*

^†^ Pinghui Zhou and Bomin Yan contributed equally to this work.

***Corresponding Authors:**

Wenrui Wang, School of Life Science, Bengbu Medical College, Bengbu 233030, China, Tel: +86-552-3175396, Email: wenrui-wang1983@163.com;

Li Zhang, Department of Plastic surgery, The First Affiliated Hospital of Bengbu Medical College, Bengbu, 233004, China, Tel: +86-552-3086021, Email: [drzhangli65@163.com](mailto:drzhangli65@163.com);

Yingji Mao, School of Life Science, Bengbu Medical College, Bengbu 233030, China, Tel: +86-552-3175396, Email: myj123@bbmc.edu.cn;

**1. Materials and MethodsMaterials**

Que (98%) and poloxam were obtained from Aladdin (China). Glyceryl stearate, cholesterol, and were purchased from Sinopharm Chemical Reagent (China). Lecithin (98%) was from Macklin (China). HA (Mw = 64 kDa) was obtained from Bloomage Biotech (China). Polyethylene glycol (PEG) (Mw = 1,450 g/mol), ε-caprolactone, and Lactide were purchased from Sigma-Aldrich (USA). Dialysis membrane (Mw= 12 kDa) was obtained from Sinopharm Chemical Reagent (China)

- 1. **Synthesis of Que-SLNs**

Synthesis of Que-SLNs was performed using the solvent emulsion diffusion method [1]. Briefly, 100 mg of poloxam was dissolved in 13 mL of ultrapure water, and 1 mL of Tween 80 was added and mixed to form the aqueous phase. The organic phase solution was obtained by adding 100 mg glyceryl stearate, 100 mg cholesterol, and 220 mg lecithin to 12 mL of acetone: chloroform (v/v=1:1) solution, followed by the addition of 20 mg Que. Both aqueous and organic phase solutions were stored at -80°C.

Subsequently, the organic solution was slowly injected into the aqueous phase at a rate of 1 mL/min under continuous mechanical stirring at 600 rpm, and the mixture was stirred for 120 min to form an emulsion. Thirteen mL of ice-cold water was added to the emulsion, and the mixture was stirred for 60 min to solidify at a low temperature (2°C). The sample was freeze-dried to obtain solid Que lipid nanoparticles. Blank lipid nanoparticles without Que were prepared using the same method. The prepared nanoparticles were sterilized by several cycles of steam sterilization and UV radiation, with confirmation that the samples were free of bacteria. Then stored at 4 °C for backup.

- 1. **Characterization of Que-SLNs**

**1.3.1 Transmission electron microscopy (TEM)**

For confirmation of the magnitude and morphology of the composed Que-SLNs nanoparticle samples, initially, the formulated nanoparticles were dissolved in distilled water and diluted to a concentration of 1 mg/mL. Thereafter, a 10 μL dilution was pipetted onto the copper network of the carbon film, and desiccated thoroughly, followed by sufficient infiltration with 2% phosphotungstic acid for negative staining. The morphological features of the nanoparticles were visualized by JEOL2010 transmission electron microscopy (JEOL, Japan).

**1.3.2 Particle size and zeta potential detection**

Further to reveal the particle dimensions and stability, Que-SLNs and blank SLNs particles were solubilized in 100 times distilled water at room temperature each, incorporated into a quartz dish, and the particle size distribution and zeta potential of the samples were assayed by Mastersizer 3000 laser particle size meter (Malvern Instruments LTD, Britain).

**1.3.3 X-ray diffraction (XRD)**

To appraise the crystallographic characteristics, the lyophilized Que, blank SLNs, and Que-SLNs pellets were weighed 20 mg each, from which the XRD spectra of the samples were subsequently detected in Rigaku Ultimate IV type X-ray auto diffractometer (Rigaku Corporation, Japan).

**1.3.4 Fourier Transform infrared spectroscopy (FTIR)**

Aiming to clarify whether encapsulation with SLNs exerts any effect on the chemical structure of the Que particles, 10 mg each of lyophilized Que, blank SLNs, and Que-SLNs solid particles were taken and blended with KBr powder, with the absorption peaks at 400~4000 cm^-1^ wavelength range of these samples were documented with IS5 FTIR spectrometer (Thermo Fisher, USA).

**1.3.5 Encapsulation efficiency and drug loading efficiency**

The 100 mg of freeze-dried Que-SLNs sample was dissolved in 10 mL of anhydrous ethanol, centrifuged at 1.5 × 10^4^ rpm at high speed, and the supernatant was diluted with an appropriate amount of mobile phase and filtered. 20 μl of the dissolved sample was injected into an Ultimate 3000 high-performance liquid chromatograph (HPLC, Thermo Fisher, USA), and the absorption peak at 360 nm was measured.

The encapsulation efficiency and drug loading capacity of quercetin were calculated using the quercetin standard curve, Where, encapsulation efficiency = amount of quercetin encapsulated in nanoparticles/total amount of quercetin drug input × 100%; drug loading = amount of quercetin encapsulated in nanoparticles/total mass of drug nanoparticles × 100%.

**1.4 Preparation of Que-SLNs@PCLA-HA scaffolds**

Initially, PCLA polymer needs to be prepared [2]. PCLA polymer was prepared by polymerizing with polyethylene glycol (PEG) (3.24 g) ( Mw = 1,620 g/mol) (Sigma-Aldrich, USA) as initiator and Sn(Oct)_2_ (0.032 g) (Sigma-Aldrich, USA) as a catalyst, followed by the addition of ε-caprolactone (CL, Sigma-Aldrich, USA) and Lactide (LA, Sigma-Aldrich, USA) for ring-opening polymerization. To be brief, PEG and Sn(Oct)_2_ were dried under vacuum at 110°C for 2 h. The reaction temperature was then rapidly lowered to 60°C and followed by the addition of CL (6.2 mL) and LA (1.84 g), which were dried under dry nitrogen protection at 60°C for 1 h and 130°C for 24 h. The obtained product was then cooled to room temperature, dissolved in chloroform, and precipitated in a 50/50 (v/v) mix of ether and n-hexane. After drying under vacuum at room temperature for 2 days, the PCLA polymer was obtained.

The HA-PCLA polymer was then prepared by coupling PCLA with the main chain of HA under an esterification reaction. HA (1 g) (M_W_ = 64,000 Da) (Creative PEGWorks, USA) was dispersed in DMF with strong stirring at the concentration of 6 mg/mL. Then, added DMAP (6 eq.) acts as a catalyst (Sinopharm Chemical Reagent Co., Ltd, China) and Boc2O (2 eq.) acts as a protective agent (Sinopharm Chemical Reagent Co., Ltd, China) and stirred for 3 h at 40°C. The above HA solution was mixed with 30 wt.% PCLA polymer solution, esterified, and allowed to react for 48 h. The solution obtained from the reaction was transferred to a dialysis membrane (MWCO= 12,000 Da) (Sinopharm Chemical Reagent Co., Ltd, China) and dialyzed with an excess of deionized water for 72 h. Centrifugation of the dialysate removed insoluble particles and the remaining solution was lyophilized to obtain HA-PCLA polymer.

**1.5** **Characterization of Que-SLNs@HA-PCLA scaffolds**

**1.5.1 Nuclear magnetic resonance (NMR)**

Characterization and analysis of the molecular structure of different hydrogels were performed employing NMR in this project. Briefly, the prepared PCLA, HA, and HA-PCLA hydrogels of 1 cm diameter and 5 mm thickness were lyophilized and ground into powder, which was fully dissolved in D_2_O at 4°C. The NMR spectra of the samples were determined by S4800 NMR (Hitachi, Japan).

**1.5.2 Fourier Transform infrared spectroscopy (FTIR)**

For further characterization of the structural properties of the different synthesized hydrogels, the aforementioned hydrogels were lyophilized into powder and their infrared spectral absorption peaks were recorded using an IS5 FTIR spectrometer (Thermo Fisher, USA) in the same way as described above.

**1.5.3 Scanning electron microscopy (SEM)**

To accurately appraise the surface morphology of the hydrogels and the impact of SLNs carrier and Que loading on the surface morphology of the hydrogels, the hydrogels were divided into four groups according to whether they incorporated Que and SLNs. The four different groups of hydrogels were lyophilized, and the lyophilized product surface was sectioned and positioned on a double-sided tape, sputtered with gold by vacuum ion coating, and the surface morphology of the microspheres was visualized with a Hitachi S4800 electron scanning microscope (Hitachi, Japan).

**1.6 Drug release and hydrogels degradation in vitro**

Based on whether SLNs carriers were used or not, the HA-PCLA hydrogels loaded with Que were divided into two groups and the cumulative release of drugs and in vitro degradation of hydrogels were examined at 37°C for comparative analysis [3]. To be brief, 2 g of 0.08 wt% Que-SLNs@HA-PCLA and Que@HA-PCLA hydrogels were precisely weighed, placed in the stainless steel cage of the drug lysimeter, immerse separately submerged in lysis cylinders filled with 0.1L phosphate buffer (0.01 M, pH 7.4) and rotated at 37°C at a rate of 100 rpm. The lysis cylinders were removed at various set time points (1, 2, 4, 7, 14, and 21 days). A 10 mL of release medium solution was drawn from the above dissolution jar, evaporated, and dried, and 0.5 mL of anhydrous ethanol was incorporated and fully solubilized, followed by high-speed centrifugation at 15,000 rpm to extract the supernatant, which was then filtered and sampled by the HPLC to detect and calculate the rate of drug release from the hydrogel.

At the same time, the remaining hydrogel in the stainless steel cage was taken out and placed in an oven at 42°C for 1 hour and then the mass was weighed precisely to calculate the degradation rate of the hydrogel.

**1.7 Isolation and culture of BMSCs**

SPF 4-week-old male Sprague-Dawley (SD) rats (Shushan Laboratory Animal Centre, Hefei, China) were sacrificed by cervical dislocation and soaked in 75% ethanol for 5 minutes. Subsequently, the skin and endothelium were cut at the leg position of the rats, and the femur and tibia were removed under sterile conditions and immersed in a sterile PBS solution containing double antibodies (penicillin 100 U/mL and streptomycin 100 U/L, PH:7.2-7.4). The muscles and connective tissue around the femur and tibia were taken and cleaned with PBS solution containing double antibodies three times. The ends of the femur and tibia were cut with ophthalmic scissors to expose the bone marrow cavity. The marrow cavity was repeatedly rinsed with pre-cooled phosphate buffer using a 5 mL sterile syringe until the marrow cavity turned white. Bone marrow fluid was collected, centrifuged, and cultured in DMEM/F-12 medium containing 10% fetal bovine serum and 1% double antibody, and placed in an incubator at 37°C, 95% humidity, and 5% CO_2_. After 24-48 hours, the cell culture medium was replaced every 3 days until cell passage was needed [4]. These cells were passed on to the third generation for subsequent experiments.

**1.8 Fabrication of rat cranial defect model**

Rats were fixed in the prone position on the experimental table, their heads were debrided and disinfected with iodophor, and a skin incision of approximately 1 cm was made in the middle of the head. The rat skull was exposed after the skin was opened by the extended incision, and a 5-mm diameter dental ring drill was sterilized and mounted on an electric drill. A 5-mm full skull defect was created by drilling two symmetric holes on both sides of the midline of the rat skull without damaging the dura mater.

**Reference**

1. Wu X, Chen H, Wu C, Wang J, Zhang S, Gao J, Wang H, Sun T, Yang YG. Inhibition of intrinsic coagulation improves safety and tumor-targeted drug delivery of cationic solid lipid nanoparticles. *Biomaterials* 2018;156:77-87.

2. Duong HTT, Thambi T, Yin Y, Kim SH, Nguyen TL, Phan VHG, Kim J, Jeong JH, Lee DS. Degradation-regulated architecture of injectable smart hydrogels enhances humoral immune response and potentiates antitumor activity in human lung carcinoma. *Biomaterials* 2020;230:119599.

3. Wei B, Wang W, Liu X, Xu C, Wang Y, Wang Z, Xu J, Guan J, Zhou P, Mao Y. Gelatin methacrylate hydrogel scaffold carrying resveratrol-loaded solid lipid nanoparticles for enhancement of osteogenic differentiation of BMSCs and effective bone regeneration. *Regen Biomater* 2021;8:rbab044.

4. Zhang Y, Li Z, Wang Z, Yan B, Shi A, Xu J, Guan J, Zhang L, Zhou P, Mao Y. Mechanically enhanced composite hydrogel scaffold for in situ bone repairs. *Biomater Adv* 2022;134:112700.


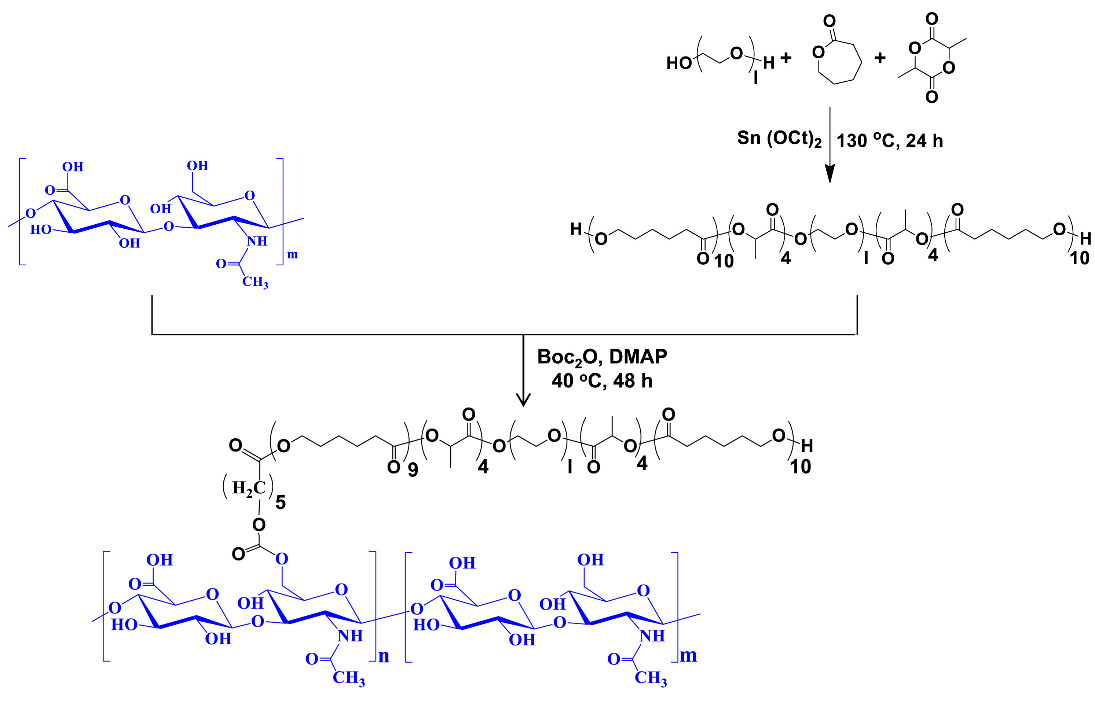


**Figure S1** Synthesis process of PCLA-HA hydrogel.

**Table S1** Quantitative RT-PCR primer sequences

| Gene | Forward primer (5′–3′) | Reverse primer (5′–3′) |
| --- | --- | --- |
| β-actin | CCCATCTATGAGGGTTACGC | TTTAATGTCACGCACGATTTC |
| ALP | GGACCCTGCCTTACCAACTC | GTGGAGACGCCCATACCATC |
| OCN | CTCAACAATGGACTTGGAGCC | GGCAACACATGCCCTAAACG |
| Runx2 | CCGAGACCAACCGAGTCATTTA | AAGAGGCTGTTTGACGCCAT |
| OPN | CCAGCCAAGGACCAACTACA | AGTGTTTGCTGTAATGCGCC |
| Col-1 | GCGGTGGTTACGACTTTGGTT | AGTGAGGAGGGTCTCAATCTG |
| OSX | CAAGAGTCGGATTCTAGGATTG | GATCAAACTTGCTGCAGGCTGCT |
